# Crystallographic texture and mineral concentration quantification of developing and mature human incisal enamel

**DOI:** 10.1038/s41598-018-32425-y

**Published:** 2018-09-27

**Authors:** Mohammed Al-Mosawi, Graham Roy Davis, Andy Bushby, Janet Montgomery, Julia Beaumont, Maisoon Al-Jawad

**Affiliations:** 1 Institute of Dentistry, Barts and the London School of Medicine and Dentistry, Queen Mary, University of London, London, E1 4NS United Kingdom; 2 School of Engineering and Materials Science, Queen Mary University of London, London, E1 4NS United Kingdom; 3 Department of Archaeology, Durham University, South Road, Durham, DH1 3LE United Kingdom; 4 School of Archaeological and Forensic Sciences, University of Bradford, Bradford, BD7 1DP United Kingdom

## Abstract

For human dental enamel, what is the precise mineralization progression spatially and the precise timing of mineralization? This is an important question in the fundamental understanding of matrix-mediated biomineralization events, but in particular because we can use our understanding of this natural tissue growth in humans to develop biomimetic approaches to repair and replace lost enamel tissue. It is important to understand human tissues in particular since different species have quite distinct spatial and temporal progression of mineralization. In this study, five human central incisors at different stages of enamel maturation/mineralization were spatially mapped using synchrotron X-ray diffraction and X-ray microtomography techniques. From the earliest developmental stage, two crystallite-orientation populations coexist with angular separations between the crystallite populations of approximately 40° varying as a function of position within the tooth crown. In general, one population had significantly lower texture magnitude and contributed a higher percentage to the overall crystalline structure, compared to the other population which contributed only 20–30% but had significantly higher texture magnitude. This quantitative analysis allows us to understand the complex and co-operative structure-function relationship between two populations of crystallites within human enamel. There was an increase in the mineral concentration from the enamel-dentin junction peripherally and from the incisal tip cervically as a function of maturation time. Quantitative backscattered-electron analyses showed that mineralization of prism cores precedes that of prism boundaries. These results provide new insights into the precise understanding of the natural growth of human enamel.

## Introduction

Precise timings and spatial progression of human enamel biomineralization are still largely unknown due to scarcity of developing human enamel specimens available for investigation. This is a crucial research question for optimizing emerging biomimetic regenerative and reparative dentistry routes.

Enamel formation is a complex protein mediated biomineralization process during which controlled fluxes of mineral-forming ions and non-collagenous matrix proteins are secreted into the extracellular space by specialized epithelial cells called ameloblasts. Mature dental enamel is generally regarded as a composite material, comprising of approximately 86% mineral, 12% water and 2% organic matrix by volume^1^ and is the most highly mineralized and hardest biological tissue in the human body^2^. The organic matrix in dental enamel consists of proteins, peptides and citric acid^1^, whilst the mineral phase is non-stoichiometric, impure hydroxyapatite (HAp) that incorporates sodium, magnesium, fluoride, hydrogen phosphate and carbonate in its lattice^3^, with the principal impurity being 2–5 *wt*% carbonate^4^, therefore throughout this paper, the term carbonated hydroxyapatite (c-HAp) will be used to refer to enamel apatite.

In developing enamel, the crystallites have been reported to appear as long plates^5^ or ribbons^6^, where the first crystallites are suggested to be 1.5 *nm* thick and 15 *nm* wide. It is debated as to whether these initial structures are crystalline octacalcium phosphates (OCP)^7,8^, or amorphous calcium phosphates (ACP) that eventually transforms into c-HAp^9,10^. In mature enamel the c-HAp crystallites assume more irregular profiles, where the majority have a roughly flattened hexagonal appearance and measure approximately 26 *nm* in thickness, 68 *nm* in width^6,11^ and with lengths that, in some cases, extend through the entire enamel thickness^12^. It is worthwhile to mention that large variations are found in the reported crystallite sizes in the literature which may be due to imaging limitations.

 At the mesoscale, c-HAp crystallites are tightly packed in an organized pattern into bundles, called prisms, approximately 2–8 *μm* in diameter, comprising approximately one thousand to several thousand^10^ c-HAp crystallites arranged in parallel arrays with their crystallographic *c*-axes predominantly oriented parallel to the long axis of the prism^13^. The cross-sectional appearance of prismatic human enamel was found to mostly resemble a “keyhole pattern” with a head and a tail component, allowing for very close packing^14,15^. The c-HAp crystallites of the “keyhole pattern” heads have a slightly different orientation to those in tails. It is a widely held view that prisms run more or less perpendicular to the enamel-dentin junction (EDJ) and the enamel surface, and that near the EDJ, the prisms divide into two groups running in slightly different directions giving rise to Hunter-Scheger bands (HSbs)^16–18^. It is important to study the precise timing and spatial progression of human dental enamel biomineralization to understand how this heterogeneous and hierarchical structure comes into existence and to inform strategies in biomimetics in order to imitate the natural process of enamel biomineralization for reparative dentistry. In order to gain a deeper insight into human dental enamel biomineralization, human enamel from the permanent dentition at different stages of development have been analyzed with a suite of techniques capable of resolving spatial and temporal changes in chemistry and structure at multiple length-scales.

Previous studies on dental enamel reported the presence of two orientation populations using synchrotron X-ray diffraction^19,20^ and lab source X-ray diffraction (XRD)^13,16,21–24^, with the precise origin of the two populations still remaining largely unknown. 2-D synchrotron X-ray diffraction (S-XRD) and X-ray microtomography (XMT) are two technique which can been used effectively to study the spatial variation in crystallite organisation and mineral content in dental enamel in health and disease^25–41^.

In this paper we aim to quantify and spatially map the direction and magnitude of organization of crystallites within the two populations at various stages of enamel development using S-XRD. This spatial quantification allows deeper understanding of the potential origins and functional significance of these two populations. Furthermore, X-ray microtomography (XMT) and quantitative backscattered electron (qBSE) imaging have been utilized in this study as complimentary techniques to assess the mineral concentration and the microstructure of dental enamel at various developmental stages.

## Results

### Enamel Mineral Concentration - X-ray microtomography

**Figure 1:**
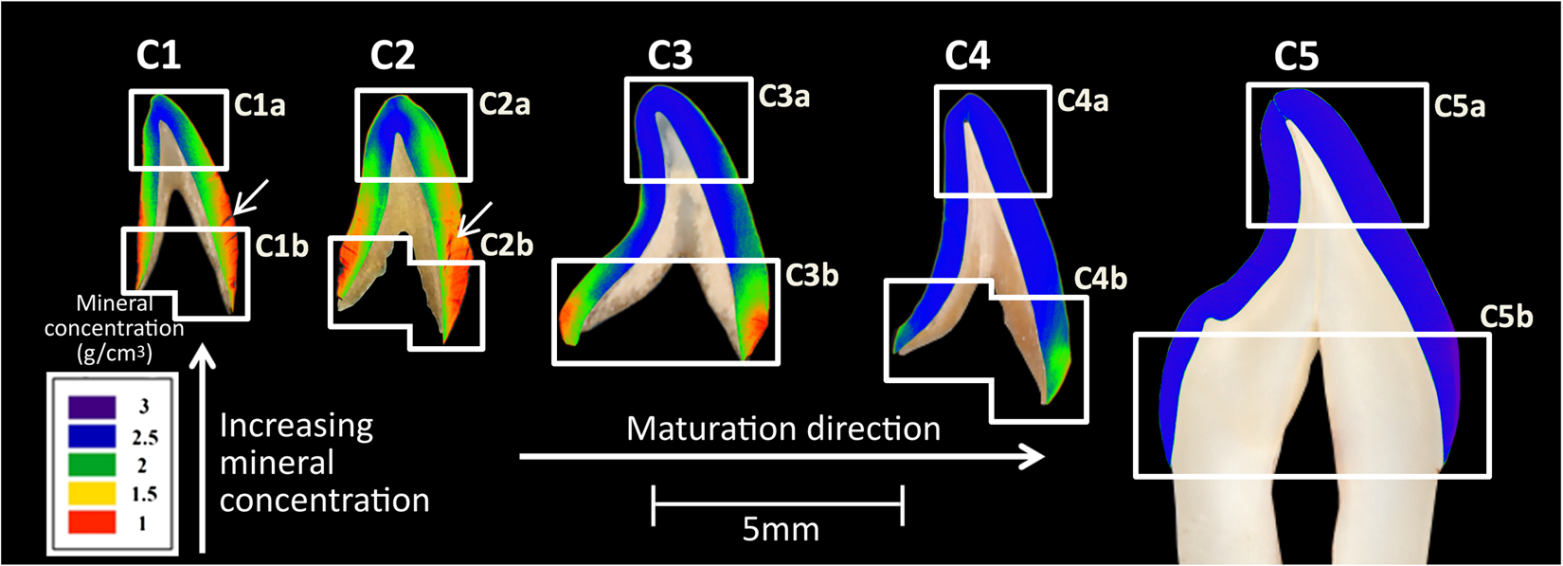
Mineral concentration distribution maps of central incisal enamel at various developmental stages. For each tooth section labial is on the right hand side.

Figure 1 displays mineral concentration maps of enamel at various developmental stages. The mineral concentration was calculated using Eq. (1). Specimens at early stages of maturation appear to possess many cracks (arrows in C1 and C2 in Fig. 1) which may be related to their lower mineral content since they are more prone to shrinkage cracking with drying. In Fig. 1, from samples C1 to C5, a general trend of increasing mineral concentration and increasing spatial uniformity of mineral concentration as a function of maturation was observed. Comparing C1 to C4 it can be seen that there is a bi-directional mineral concentration “front” that starts at the cusp tip and at the EDJ and travels towards the cervical end and the enamel surface until the mineral concentration is uniform in the fully mature tooth (Fig. 1 C5).

**Figure 2:**
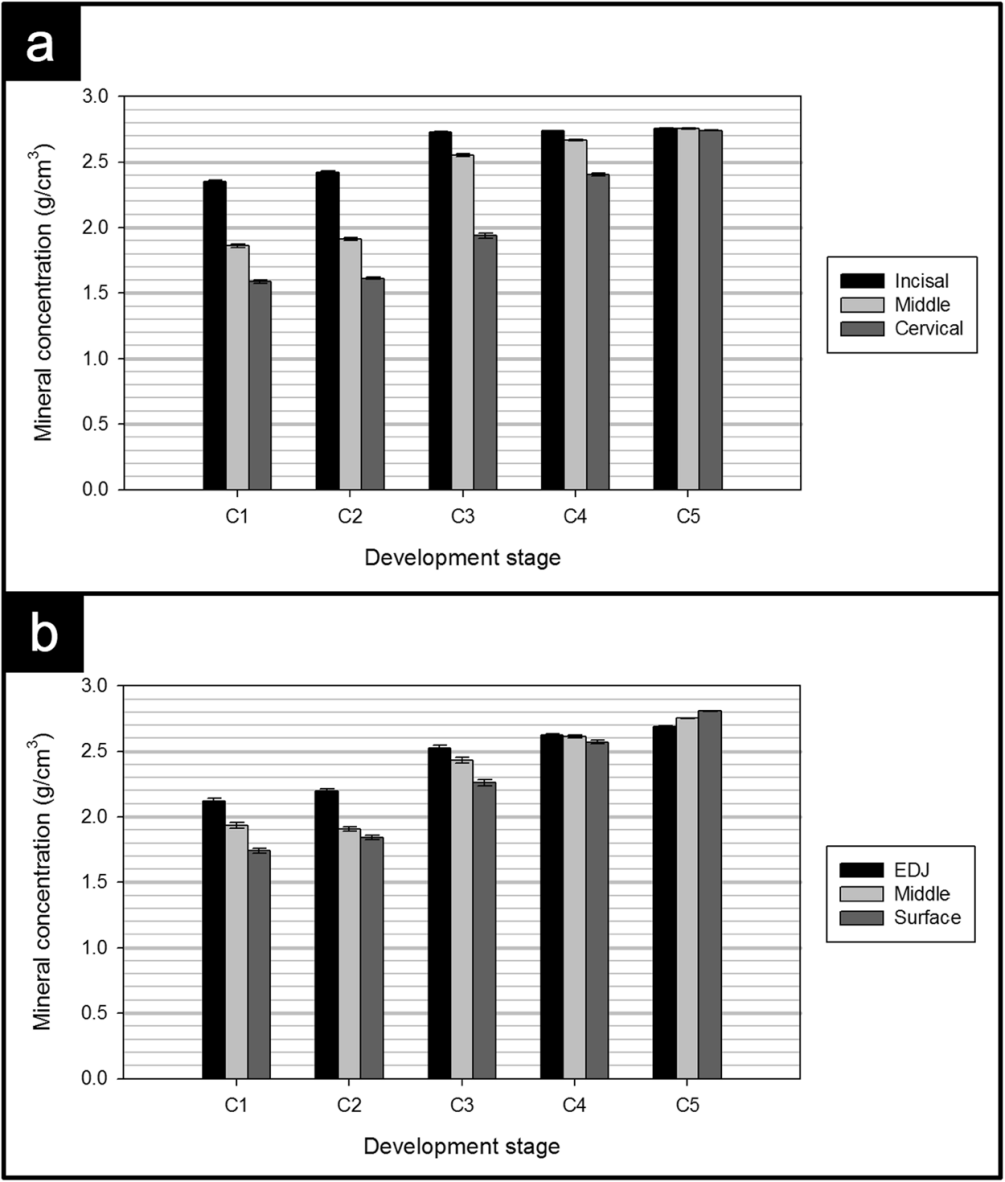
(**a**) Vertical and (**b**) horizontal distribution of mineral concentration of central incisal enamel at various developmental stages.

Quantitatively assessing the variation in mineral concentration vertically, i.e. from the cusp toward the cervical end, developing enamel in specimens C1, C2, C3 and C4 had substantially higher average mineral concentration near the incisal edge (such that in Fig. 1: C1a = 2.35 *gcm*^−3^, C2a = 2.42 *gcm*^−3^, C3a = 2.73 *gcm*^−3^, C4a = 2.74 *gcm*^−3^) compared to the cervix of the tooth (such that in Fig. 1: C1b = 1.59 *gcm*^−3^, C2b = 1.61 *gcm*^−3^, C3b = 1.94 *gcm*^−3^, C4b = 2.4 *gcm*^−3^). In contrast, fully developed enamel in specimen C5 displayed a more uniform mineral concentration when comparing the incisal (Fig. 1: C5a = 2.76 *gcm*^−3^) and cervical (Fig. 1: C5b = 2.74 *gcm*^−3^) ends. For comparison, Fig. 2a shows the vertical variations and Fig. 2b shows the horizontal variations in mineral concentration for the 18 regions displayed in Fig. 9b,c.

Further analysis showed that in developing enamel the mineral concentration was higher near the EDJ (such that in Fig. 1 C1 = 2.12 *gcm*^−3^, C2 = 2.20 *gcm*^−3^, C3 = 2.53 *gcm*^−3^, C4 = 2.63 *gcm*^−3^) compared to the enamel surface (such that in Fig. 1 C1 = 1.74 *gcm*^−3^, C2 = 1.84 *gcm*^−3^, C3 = 2.26 *gcm*^−3^, C4 = 2.57 *gcm*^−3^). Conversely, in the fully developed enamel (sample C5), the highest mineral concentration was near the surface (2.81 *gcm*^−3^) and the lowest was near the EDJ (2.69 *gcm*^−3^) (Fig. 2b). The standard error of the mean values of the above-mentioned mineral concentration measurements were found to be less than 0.02 *gcm*^−3^. It was reported previously that the systematic error in the quantification of pure HAp was in the order of 1%^42^.

### Crystallite organization and orientation - 2-D synchrotron X-ray diffraction

#### Texture direction

**Figure 3:**
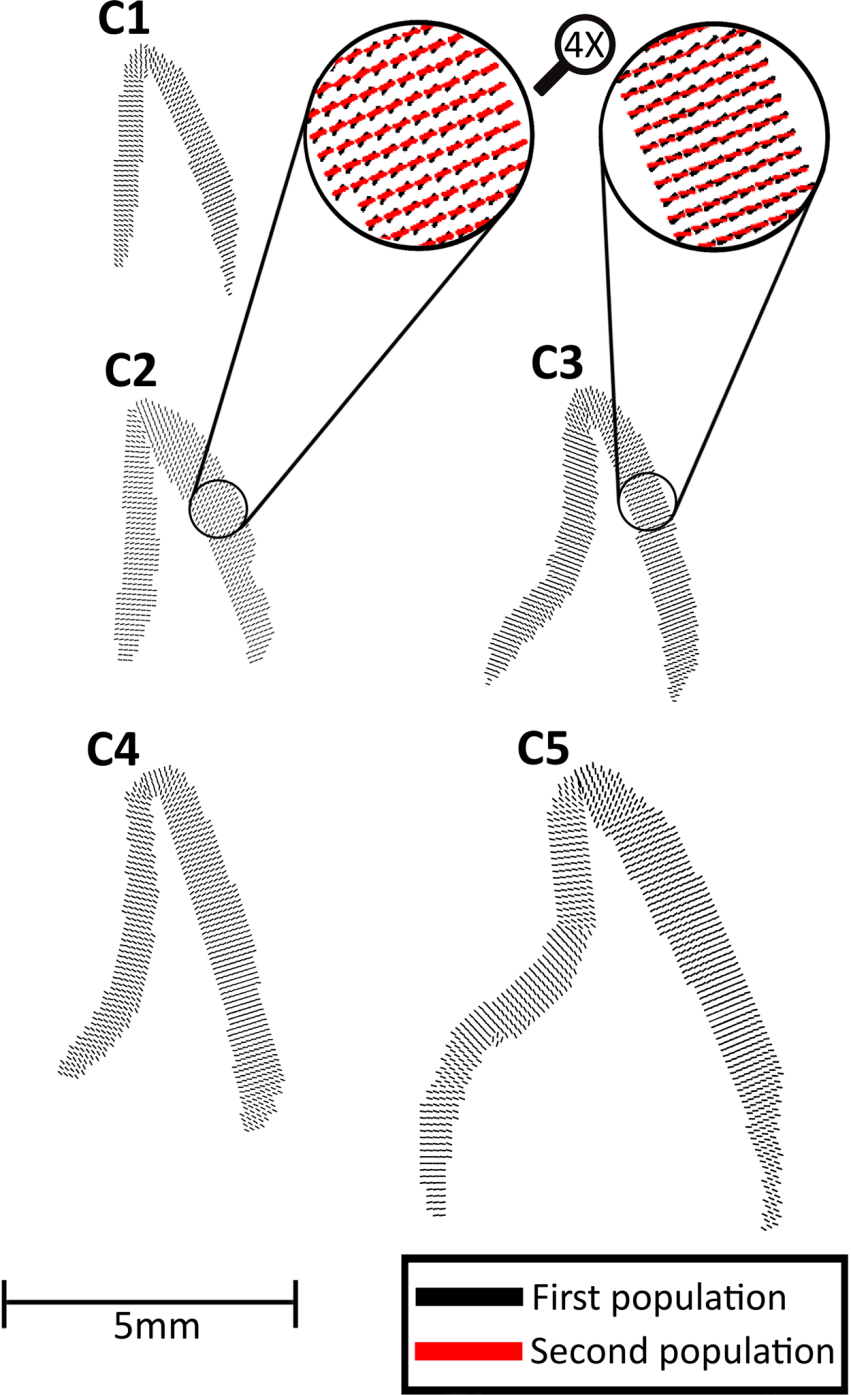
Texture direction maps of crystallites from the first orientation population in enamel at various developmental stages. Two regions were selected and magnified to show the texture directions of crystallites from both orientation populations. For each tooth section the labial surface is on the right hand side.

S-XRD revealed that within one probed region, two populations of crystallite orientations coexist simultaneously with an angular separation of 20–50°. Fig. 3 displays the texture direction maps of the first population of c-HAp crystallites of enamel at various developmental stages. For clarity, two regions were selected and magnified (inserts in Fig. 3) to show the texture directions of both populations in samples C2 and C3. Further, it can be seen from Fig. 3, that the long axes of c-HAp crystallites were approximately perpendicular to both the enamel surface and the EDJ. Moreover, the crystallites of both the most mature of the developing samples (sample C4) and the fully-developed sample (sample C5) were directed more gingivally near the cervical parts of the crowns. This phenomenon was not present in the three least developed samples (samples C1, C2 and C3), where the direction of crystallites was approximately horizontal cervically. In the central part of the teeth, crystallites were found to be somewhat horizontal in all developmental stages. Near the incisal tip, the directions of crystallites in all developmental stages were found to change gradually to an increasingly oblique direction until they were almost vertical.

#### Population percentage

**Figure 4:**
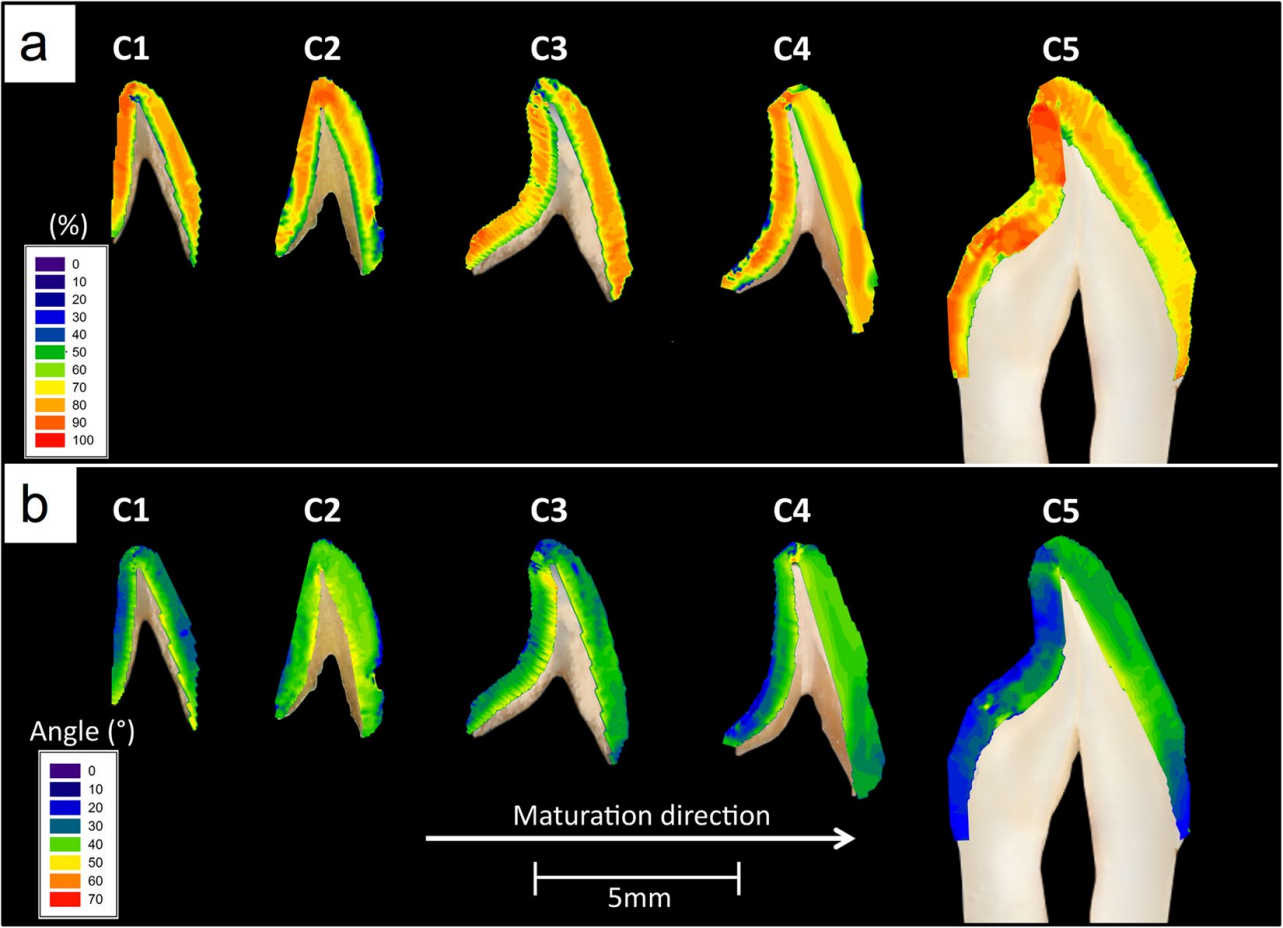
(**a**) The percentage of crystallites belonging to the first orientation population and (**b**) the angle between the two orientation populations in central incisal enamel at various development stages. For each tooth section the labial surface is on the right hand side.

Peak intensities from the azimuth of the (002) Bragg reflection correlate with the quantity of *c*-planes in the c-HAp crystallites satisfying the diffraction condition. This allows for predicting the relative percentage of the two orientation populations in each diffraction pattern. The results from Fig. 4a revealed that the first orientation population is the dominant population in all samples (*C*1 = 73.9%(*SD* = 16.0%), *C*2 = 64.3%(*SD* = 15.9%), *C*3 = 69.9%(*SD* = 14.8%), *C*4 = 70.2%(*SD* = 11.4%) and *C*5 = 73.4%(*SD* = 10.7%)).

#### Angle between orientation populations

Figure 4b displays the spatial distribution of angular separation for the five samples. It was found that the angular difference between the two orientation populations of the samples at the various development stages increased from the enamel surface towards the EDJ.

#### Texture magnitude

**Figure 5:**
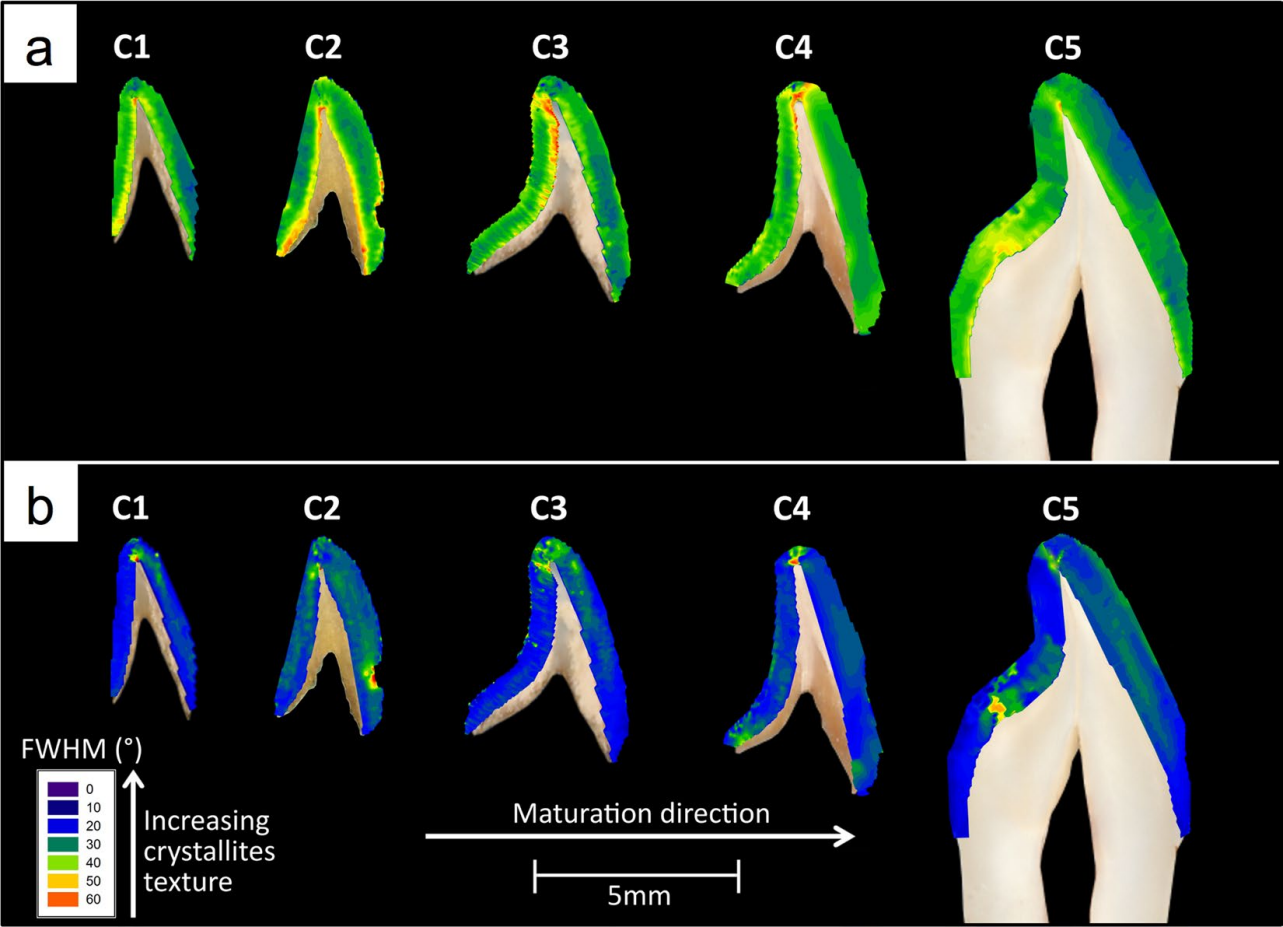
Texture magnitude distribution of crystallites from the (**a**) first and (**b**) second orientation populations in central incisal enamel at various development stages. For each tooth section labial is on the right hand side.

In addition to the considerable local variations in crystallites texture magnitude, it was observed that in all assessed samples, the first orientation population (Fig. 5a) had lower texture magnitude than the second population (Fig. 5b).

In sample C1, the crystallites from the first population possessed lower texture magnitude along the EDJ in both palatal and labial enamel. Overall, the texture magnitude of crystallites in labial enamel was found to increase from the incisal tip towards the cervical part of the tooth. Conversely, in palatal enamel, the texture magnitude of crystallites was found to decrease from the incisal tip towards the cervical part of the tooth. Moreover, the crystallites in palatal enamel were found to have lower texture magnitude than those in labial enamel (Fig. 5aC1).

The crystallites from the second orientation population displayed a low degree of texture at a region near the incisal tip, extending to one third through the bulk of the labial enamel. Furthermore, the texture magnitude was found to decrease from the cervical part towards the incisal tip of labial enamel. In palatal enamel, the crystallites displayed more homogeneous texture magnitude than those found in labial enamel. Further, the crystallites in palatal enamel had higher texture magnitude than those in labial enamel (Fig. 5bC1).

In sample C2, the crystallites from the first orientation population had lower texture magnitude along the contour of the EDJ than those near the enamel surface in both palatal and labial enamel. In labial enamel, the degree of texture was found to increase from cervical part towards the incisal tip of the tooth. Moreover, it was revealed that the highest texture magnitude was in the bulk of the tooth (the area between the EDJ and the enamel surface). In palatal enamel, the central region was found to contain crystallites with higher texture magnitude than those in the upper and lower parts of the tooth. On average, the crystallites from the first orientation population were found to have lower texture magnitude in labial enamel than those in palatal enamel (Fig. 5aC2).

The crystallites of the second orientation population in C2 were found to have lower texture magnitude than those of the least developed sample (sample C1). The texture magnitude was found to increase from the incisal tip towards the cervical part of the tooth. Additionally, an area with crystallites having considerably low texture magnitude was observed around a chipped section of the tooth in labial enamel. Further, the crystallites in palatal enamel had higher texture magnitude than those in labial enamel (Fig. 5bC2).

In sample C3, the texture magnitude of crystallites from the first orientation population in both palatal and labial enamel was found to increase from incisal tip towards the cervical part of the tooth. In labial enamel, the crystallites in the top half of the tooth had lower texture magnitude along the EDJ compared to those along the enamel surface. However, the crystallites in the bottom half of the tooth displayed higher texture magnitude along the EDJ compared to those along the enamel surface. In palatal enamel, it was observed that the crystallites along the EDJ in the top half of the tooth had a considerably lower degree of texture than those along the enamel surface. However, the crystallites along the EDJ and the enamel surface in the bottom half of the tooth displayed a similar degree of texture, with crystallites having higher texture magnitude in the enamel bulk. Further, crystallites in palatal enamel were found to display on average lower texture magnitude than those in labial enamel (Fig. 5aC3).

The crystallites from the second orientation population in C3 displayed low texture magnitude in a region near the incisal tip. In labial enamel, the texture magnitude of crystallites was found to decrease from cervical part towards the incisal tip of the tooth. However, in palatal enamel, the texture magnitude of crystallites was found to be more homogeneous throughout and on average higher than that of labial enamel (Fig. 5bC3).

In sample C4, the crystallites of the first orientation population near the dentin horn were found to display low texture magnitude. Furthermore, it was observed that crystallites in the bulk of palatal and labial enamel displayed the highest texture magnitude, whereas the areas near the EDJ and enamel surface contained less textured crystallites. Additionally, the results showed a region of relatively lower degree of texture near the cervical ends of both palatal and labial enamel (Fig. 5aC4).

Crystallites from the second orientation population in C4 were found to possess a low degree of texture in a small area extending from the dentin horn towards the tip of the tooth. Moreover, areas consisting of crystallites with low texture magnitude could be seen near the cervical ends of palatal and labial enamel. Further, crystallites along the EDJ of labial enamel had a higher degree of texture than those along the enamel surface (Fig. 5bC4).

In sample C5, the fully mature specimen, the texture magnitude of crystallites belonging to the first orientation population was found to increase from the cervical part towards the incisal tip of the tooth in both labial and palatal enamel. In labial enamel, it was observed that crystallites along the EDJ displayed lower texture magnitude than those along the enamel surface. In palatal enamel, crystallites along the EDJ and the enamel surface in the top half of the tooth were found to have similar degree of texture. However, crystallites along the EDJ in the lower half of palatal enamel displayed lower texture magnitude than those along the enamel surface. Overall, crystallites in labial enamel were observed to have a considerably higher degree of texture than those in palatal enamel (Fig. 5aC5).

Crystallites from the second orientation population in C5 in labial and palatal enamel were found to have a higher degree of texture near both the upper and cervical ends than those in the central part of the tooth. In labial enamel, crystallites along the enamel surface in the top half of the tooth had considerably lower texture magnitude than those along the EDJ. However, the crystallites along the EDJ and the enamel surface in the lower half of labial enamel were found to display similar degree of texture (Fig. 5bC5).

### Microstructures - Quantitative backscattered-electron imaging

**Figure 6:**
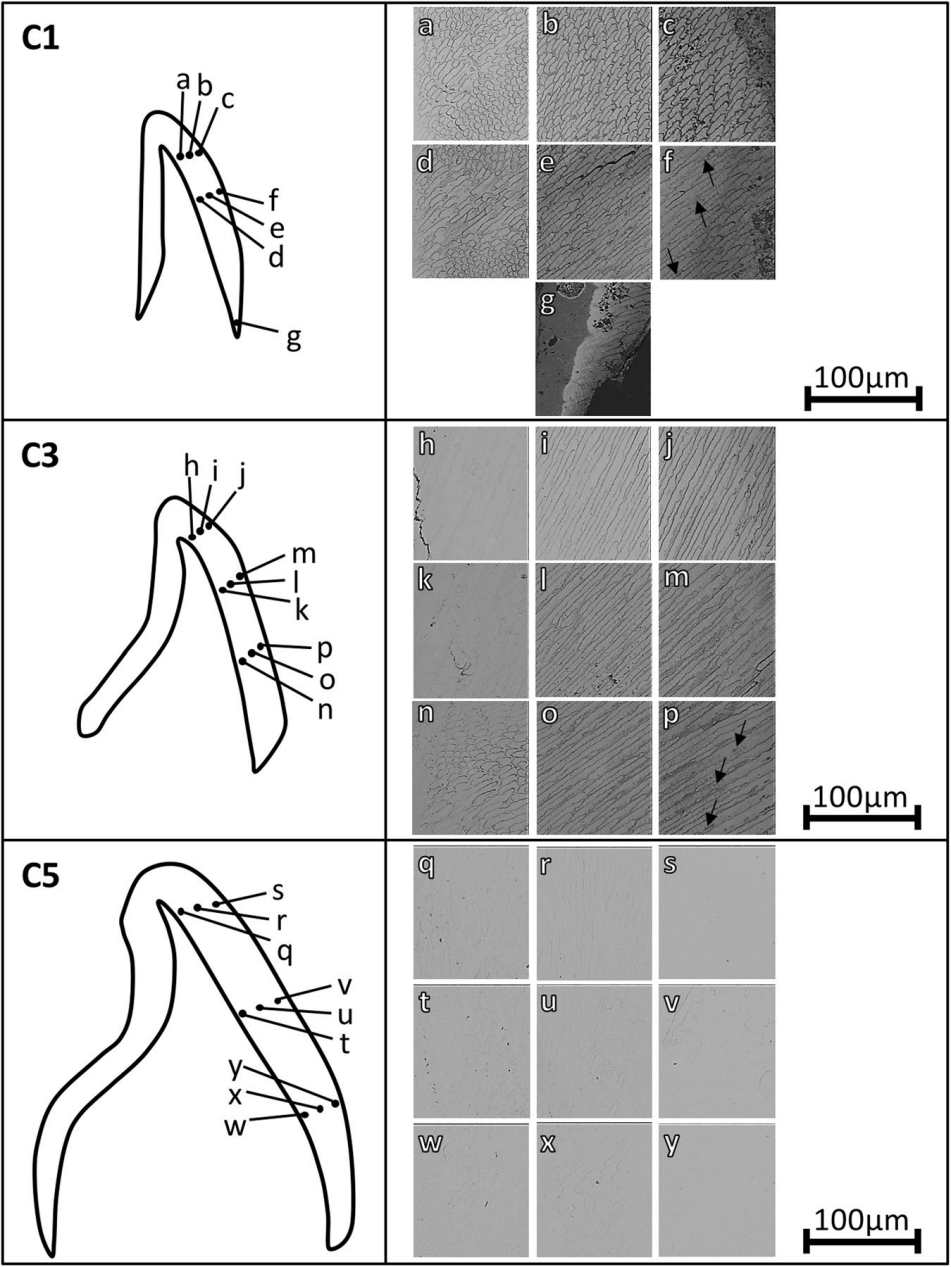
qBSE images at various locations of central incisors at **(a**–**g**) early-, **(h**–**p**) mid- and **(q**–**y**) full- development. The arrows in images (**f**) and (**p**) indicate hypomineralized intra-prismatic regions. For each labial section the enamel surface is on the right hand side.

qBSE imaging results showed that prisms run approximately perpendicular to enamel surface in all developmental stages (Fig. 6).

In sample C1, the enamel prism boundaries were found to be prominent throughout the thickness of the crown (Fig. 6a–g). Furthermore, near the EDJ, the top (Fig. 6a) and central (Fig. 6d) parts of the crown displayed two groups of prisms with two distinct morphologies. One group of prisms was longitudinally elliptical in shape (with the long axes approximately perpendicular to the EDJ), whereas the other group was somewhat circular. This phenomenon continued through the bulk of enamel in the central part of the tooth (Fig. 6e). However, the enamel prisms in the cervical part of the tooth (Fig. 6g) did not display major variations in morphology, with most prisms being somewhat elliptical in shape. Moreover, the enamel near the surface in the central (Fig. 6f) and cervical (Fig. 6g) part of the tooth displayed dark areas within the developing prisms i.e. away from the prism boundaries (arrows in Fig. 6f). In the top part of the crown, the prisms in the enamel bulk (Fig. 6b) and near the surface (Fig. 6c) had similar morphologies with distorted keyhole shape and were shorter than those in the central part of the tooth (Fig. 6e,f). Furthermore, groups of prisms in the enamel bulk (Fig. 6e) and near the surface (Fig. 6f) in the central parts of the tooth displayed slight difference in direction.

In sample C3, the enamel prism boundaries near the EDJ in the top part of the crown (Fig. 6h) could not be identified, becoming more prominent in the enamel bulk (Fig. 6I,l) and near the enamel surface (Fig. 6j,m). However, near the lower part of the tooth, the prism boundaries were prominent throughout the thickness of enamel (Fig. 6n–p). Moreover, the enamel near the surface in the cervical part of the tooth (Fig. 6p) displayed dark areas within the bulk of the developing prisms (arrows in Fig. 6p). Furthermore, near the EDJ, the central (Fig. 6k) and cervical (Fig. 6n) parts of the crown displayed two groups of prisms with two distinct morphologies. One group of prisms was longitudinally elliptical in shape (with the long axes approximately perpendicular to the EDJ), whereas the other group was somewhat circular. Prisms in the bulk of enamel (Fig. 6i,l and o) and near the enamel surface (Fig. 6j,m and p) were somewhat elliptical in shape.

In the fully developed enamel (sample C5), the prism boundaries were more difficult to distinguish compared to those in developing enamel. In the enamel near the EDJ at the top (Fig. 6q) and central (Fig. 6t) parts of the tooth, two groups of prisms were identified, one with elliptical appearance (with the long axes approximately perpendicular to the EDJ) and the other with a somewhat circular morphology. This phenomenon was found to continue through to the bulk of enamel at the central (Fig. 6u) and cervical (Fig. 6x) parts of the tooth. However, the prisms in the enamel bulk at the top part of the crown (Fig. 6r) were elliptical in shape with their long axes approximately perpendicular to the enamel surface. The prism boundaries could not be identified in the enamel near the surface (Fig. 6s,v and y).

## Discussion

The XMT results, as shown in Fig. 1, indicated that there was a progressive increase in the amount of mineral in human enamel as a function of maturation, agreeing with previous studies using Fourier transform infrared spectroscopy (FTIR) on pig enamel^43^ and polarized light analysis^44^ and XMT on human enamel^29^. Upon closer examination, the results showed that on average the mineral concentration was highest at the incisal tip in developing enamel, decreasing away from the incisal tip cervically. This suggests that biomineralization proceeds cervically from the tip of the tooth, agreeing with previous studies using chemical analysis to assess developing human enamel^45^ and corresponding microradiographic and hardness studies^46^ and polarized light analysis of developing human enamel^44^.

Furthermore, the fully developed enamel (sample C5) was found to have a much more uniform mineral concentration distribution compared to developing enamel (Fig. 1). The mean mineral concentration of the fully mature enamel was 2.75 *gcm*^−3^ ± 0.01 *gcm*^−3^, which is in agreement with a previous XMT study on human molars by Simmons *et al*.^29^ yielding a value of 2.73 *gcm*^−3 ^^29^. Further analysis of the fully developed enamel showed that the enamel at the upper part of the tooth had very similar mineral concentration (2.76 *gcm*^−3^ ± 0.01 *gcm*^−3^) to cervical enamel (2.74 *gcm*^−3^ ± 0.01 *gcm*^−3^) (Fig. 2a) which agrees with the trend seen in previous studies on mature enamel using density gradient measurements^47^, microradiography^48^ and XMT^29^. Moreover, Fig. 2b showed that, unlike mature enamel, developing enamel possessed higher mineral concentration near the EDJ than the tooth surface. This phenomenon was reported in various studies assessing developing human enamel, using soft X-ray analysis^49^, polarized light microscopy^44^, fluorescence microscopy^50^, microradiography^46,51–54^ and XMT^29^.

Previous work on developing bovine enamel revealed that the highest mineral concentration was near the EDJ until maturation begins^55^. However, it was reported that the crystallites of developing human enamel are thinner at the EDJ than the crystallites close to the enamel surface^5^. It was also observed that there is a decrease in the number of crystallites in mature enamel from the enamel surface towards the EDJ which suggests that crystallites near the EDJ may undergo fusion^6^. It is also believed that the prism diameter increases from the EDJ towards the enamel surface in order to accommodate the larger outer radius^18,56^. The findings of this study together with the above-mentioned supporting literature, suggest that enamel biomineralization progresses from the EDJ towards the surface. The reason why crystallites grow from inside to outside following the sequence of protein removal is, most probably, to prevent sealing the surface as ions enter from the surface ameloblast layer^57^.

The above mentioned results suggest that mineralization progression is bi-directional from the incisal edge to the cervix and from the EDJ to the enamel surface. This is in good agreement with a recent study reporting that mineralization processes start beneath the future tip and then proceed cervically and from EDJ to the tooth surface^58^. These results serve as a confirmation that the mineral content in the archaeological samples follow the expected route of mineralization, where the highly mineralized region continues to advance toward the enamel surface and the tooth cervix as a function of maturation. Therefore, the results obtained from such samples indicate that burial for extended periods has had little effect on the mineral content of enamel and gives further validity to the S-XRD results.

It has been established previously that the long axes of c-HAp crystallites in dental enamel are approximately perpendicular to the EDJ and to the enamel surface^25,29,59–62^. Boyde and Fortelius^63^ suggested that prisms with their long axes perpendicular to the enamel surface offer the greatest resistance to abrasion during function. The results in Fig. 3 showed that this feature was persistent in the two populations of crystallites regardless of the developmental stage, indicating that the initial preferred growth directions of apatite crystallites persist from early through to full maturation. This was confirmed with qBSE which revealed that the prisms with the longitudinally elliptical morphology had their long axes approximately perpendicular to the enamel surface (Fig. 6). This is in agreement with the current understanding that prisms run approximately perpendicular to the enamel surface^25,29,59–62^. According to Boyde^64^, perpendicularity to enamel surface, allows for a more efficient packing of crystallites, maximizes their strength and bendability and enhances their wear resistance capabilities. This gives direct evidence to previous assumptions that crystallite orientations are defined early in development^9,65^. This also supports the early assumption by Boyde^14^ that crystallites, once formed, tend to continue growing in the same long axis (*c*-axis) direction. Furthermore, these findings serve as indirect evidence that any post-mortem diagenesis in our samples has not affected the structure at the micro- nano- and sub-nano- length scales.

Using 2-D S-XRD has allowed us to understand that within one probed region, crystallites group along two main directions with respect to their *c*-axes with an angular separation between the two main directions varying spatially across the enamel crown in a range of 20–50° (Fig. 4b). Although the presence of two orientation populations has been reported in other studies on dental enamel using S-XRD^19,20^ and lab source XRD^13,16,21–24^, here we have been able to quantify and spatially map the direction and magnitude of organization of crystallites within each population. This spatial quantification allows deeper understanding of the potential origins and functional significance of these two populations.

A single 2-D diffraction pattern contains information averaged over several hundred prisms (average prism diameter is 5 *μm*), therefore, the two orientations observed could be the result of orientation variation between the “heads” and “tails” of the prismatic keyhole pattern, or due to prism decussation occurring throughout the probed thickness of the sample. It has been reported using scanning electron microscopy (SEM) on human enamel that not all crystallites within a single prism run parallel to the prism long axis. Some crystallites have their long axes orientated toward the keyhole tail fanning out in a range of 0–70° in relation to the prism long axis^15,66^. Thewlis^21^ suggested that the two crystallite orientation populations observed using lab source XRD represent differences in orientation between crystallites in the head and tail of the keyhole pattern. In contrast, we observe two populations with discrete orientations (Fig. 12), not a gradual “fanning” of crystallites. In addition Glas^13^ observed the two orientation populations while assessing a 10 *μm* thick slice of human enamel, therefore, it is unlikely that two populations arise as a result of overlapping prisms through the sample volume.

A study on bovine enamel speculated that the two orientation populations do not exist within a single prism, but are the result of probing two groups of prisms, or prisms from different regions of the enamel^67^. The two orientation populations observed could be the result of probing two zones of HSb simultaneously. It was previously shown that there is relatively little crossing of prisms in the outer quarter of human enamel where prisms run approximately parallel to one another, then suddenly twist as they approach middle and inner enamel and divide into two groups deviating in opposite orientations corresponding to the two zones of the HSb^16,68^. These are the features that can be observed in detail in Fig. 4b, where the angular difference between the two orientation populations decreases from the EDJ peripherally.

Furthermore, it can be seen in Fig. 4b that in the fully developed enamel (sample C5), the angular difference between the two orientation populations is lowest near the cervical regions, which according to Lynch *et al*.^69^, have little or no prism decussation, most likely due to the fact that enamel in these regions is not subjected to high loading forces. Moreover, Fig. 4b shows that samples in all developmental stages (with the exception of sample C2) possessed a small region with low angular difference near the incisal tip, a region that is believed to contain no HSb^69^. Lynch *et al*.^69^ suggested that the absence of prism decussation at the incisal tip allows for rapid abrasion of this region to expose the HSb-rich region underneath in order for the enamel edges to be kept sharp leading to enhanced mastication. In further support of this evidence-based hypothesis, Macho *et al*.^18^ developed a 3-D model based on processes governing enamel formation in humans and dogs where it can be seen that the prism decussations decrease from the EDJ towards the enamel surface.

In all samples, the first orientation population of crystallites (Fig. 5a) had a considerably lower degree of preferred orientation (texture) than the second orientation population (Fig. 5b). It has been previously reported that mature enamel possess higher texture magnitude near the cusps of premolars^25–27^, molars^29,62^ and incisors^62^. Further, it was found that the Young’s modulus and hardness of mature human enamel decrease from the cusp cervically^70^. This suggests that crystallite texture magnitude is somewhat linked to hardness and Young’s modulus which are in turn related to wear resistance. Moreover, it is believed that regions with lower crystallite alignment displayed lower hardness and Young’s modulus values^70.^

 It was observed in a study assessing 160 human incisors, canines, premolars and molars, that the most axial surface possesses the highest mean HSb packing densities compared to other regions of the tooth^69^. Our results from mature enamel (sample C5) showed that the texture magnitude of crystallites belonging to the first population decreased from the incisal tip towards the cervical part of the tooth, agreeing with the above-mentioned studies (Fig. 5aC5). However, the second population of crystallites at the incisal and cervical parts of mature enamel displayed similar texture magnitude values, which were higher than those at the central part of the tooth (Fig. 5bC5).

Further, Lyon and Darling^66^ and Glas^13^ who assessed mature enamel using polarized light microscopy (PLM) and XRD respectively, suggested that the crystallites near the EDJ had the poorest texture magnitude. Furthermore, using S-XRD to assess human mature premolars^25–27^ and molars^29^, it was reported that crystallites near the EDJ displayed the lowest degree of orientation which is the site where the first enamel crystallites are believed to form^55^. Although this was the case in our assessment of mature labial enamel regarding the first population of crystallites (Fig. 5aC5), the second population of crystallites along the EDJ had higher degree of texture than those along the enamel surface (Fig. 5bC5). This was also the case in our results from developing enamel (samples C1, C2, C3 and C4), where the crystallites of the first orientation population were found to display lower degree of texture along the contour of the EDJ than those along the enamel surface (Fig. 5a).

Simmons *et al*.^29^ also reported that the sample in earliest development displayed a relatively homogeneous texture distribution. Our results from the least developed enamel (sample C1) showed that the texture magnitude of the crystallites from the second population was more homogeneous than the other specimens (Fig. 5b) agreeing with Simmons *et al*.^29^. However, the first population of crystallites displayed similar local variations in texture magnitude in all analyzed teeth (Fig. 5a). Furthermore, Simmons *et al*.^29^ reported that texture magnitude decreased as a function of maturation in molar enamel. However, the results from both the first (Fig. 5b) and second (Fig. 5b) orientation population showed that the texture magnitude of the crystallites in incisal enamel does not follow a clear trend as a function of maturation.

It is interesting to note that previous studies assessing dental enamel texture properties are in agreement with our results regarding the crystallites of the first population at the EDJ^13,25–27,29,66,71^ and at the upper part of the tooth^25–27,29,62^. However, our observed texture variations of the second population of crystallite orientations have not been reported previously and are considerably different to that of the first population. It is also worth mentioning as can be seen in Fig. 4a that the first population is the dominant one and is most likely the population analyzed in the above-mentioned studies and perhaps the second population in the past has not been as significant.

We observed differences in the organization of crystallites when comparing the labial and palatal side of the crown. The first population of crystallites in mature labial enamel possessed a higher degree of texture than those in palatal enamel. Conversely, the second population of crystallites in labial enamel at the various developmental stages possessed lower texture magnitude than those in palatal enamel. It is evident from our results of both developing and mature enamel that, in most cases, texture magnitude of one population is inversely related to the other. That is, generally speaking, when the texture magnitude of one population is higher in a region; the texture magnitude of the other population will be lower at that same region and vice versa. This brings us to the conclusion that previous investigations did not just average the two populations but completely overlooked one of the populations.

It has been reported that in developing enamel, the enamel mineral concentration near the EDJ is higher than that near the surface^29,44,46,49–54^, suggesting that mineralization starts from the EDJ peripherally. qBSE images showed that the least mineralized regions of the developing teeth (C1 and C3 in Fig. 6f,g and p) had dark prism boundaries and heterogeneous prism cores with some light and some darker areas within the prism cores. The more mineralized regions of developing teeth (C1 and C3 in Fig. 6a,i) also had dark prism boundaries, however the prism cores were uniformly mineralized (no dark areas observed) indicating at this stage the prism cores are fully mineralized whilst the prism boundaries are not. Prism boundaries are believed to be organic-rich discontinuities in the overall structure between different orientations of crystallites^64^. According to Boyde^64^, the organic material is not concentrated in those locations at the initiation of enamel formation but it is concentrated there during the re-mobilization of the matrix during the maturation of enamel. The prism boundaries near the EDJ at the top part of the mid-developed enamel (sample C3) (Fig. 6h) could not be easily distinguished, becoming more prominent when moving peripherally and cervically across the crown. The results presented here therefore suggest that prism cores were mineralized prior to prism boundaries. This further supports the theory that prisms calcify from the center peripherally^46^. The reason behind this can be that during enamel development the prism tails become the route taken by the mineralizing ions to reach the centers of prisms to calcify them. Once the prism centers calcify, those boundaries are then sealed by mineralizing later^46^. Further, near the central (Fig. 6k–m) and lower (Fig. 6n–p) parts of the tooth in mid-development (sample C3), the prism boundaries were found to be prominent throughout the thickness of enamel. It was reported previously, that the lower parts of developing teeth has lower mineral concentration and hence are less developed than the upper regions of the crown^44–46,54,72^, confirming that mineralization progresses from the top of the crown cervically. This explains the prominence of prism boundaries at this region and confirms the hypothesis stating that prism cores mineralize prior to prism boundaries.

Furthermore, various qBSE images displayed two groups of prisms with two distinct morphologies. One group of prisms was found to be elliptical in shape, whereas the other group was somewhat circular. The two groups of prisms were visible near the EDJ at the upper (Fig. 6a) and central (Fig. 6d) parts of the least developed enamel (sample C1) and at the central (Fig. 6k) and lower (Fig. 6n) parts of the enamel at mid-development (sample C3). The two groups of prisms could also be seen near the EDJ at the top (Fig. 6q) and central parts (Fig. 6t) of mature enamel (sample C5). This phenomenon was found to continue through to the bulk of mature enamel (sample C5) at the central (Fig. 6u) and cervical (Fig. 6x) parts of the tooth. However, prism boundaries could not be identified near the surface of mature enamel (Fig. 6s,v and y). The two prism morphologies observed near the EDJ are in agreement with a number of studies who suggested that towards inner enamel, prismatic bundles cross-over or decussate in a stepwise manner, giving rise to HSb^16–18,64,69,73–77^. It was hypothesized that prism decussation gives rise to unequal wear rates^64^ and prevents crack propagation while strengthening the enamel in respect to the horizontal tension forces^78^.

At the top part of the least developed sample (sample C1), the prisms at the enamel bulk (Fig. 6b) and near the surface (Fig. 6c) had similar morphologies with distorted keyhole shape (Fig. 6e,f). This morphology was observed in a model constructed by Meckel *et al*.^15^, who reported it to appear when the plane of the model surface deviates by 5° from the surface. However, in the mid-developed sample (sample C3), the prisms at the bulk of enamel (Fig. 6i,l and o) and near the enamel surface (Fig. 6j,m and p) were somewhat elliptical in shape. Furthermore, in the fully developed enamel (sample C5) the prisms at the enamel bulk at the top part of the crown (Fig. 6r) were elliptical in shape. The similarity in prism morphologies near the surface reported in the current study agrees with the accepted understanding that the outer enamel pattern of mammals consists of groups of prisms running in similar directions that suddenly divide into two groups running in two different directions as they approach the EDJ^16–18,69,77^.

**Figure 7:**
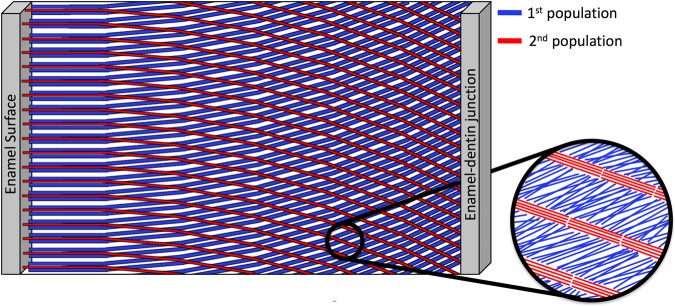
A proposed model representing the texture magnitude and direction of the two orientation populations as they span the enamel thickness. The thicker the lines, the lower the texture magnitude and the higher the number of lines, the higher the population percentage.

S-XRD revealed that crystallites were found to group into two main directions with respect to their long axes. The angular separation between the two populations was found to be higher near the EDJ compared to the enamel surface (Fig. 4b). qBSE images revealed that prisms near the enamel surface were approximately parallel to one another, then divide into two groups running at two different directions as they approach the EDJ (Fig. 6). This is suggested to be caused by secretory ameloblast cells moving laterally, left or right parallel with neighboring cells, where transverse rows of cells move in opposite directions^10,55^. Therefore, we propose that the two orientations observed may correspond to the crystallites within the two groups of the decussating prisms. Figure 7 displays a proposed model based on the gathered data, displaying the two crystallite orientation populations as prisms decussate from the enamel surface towards the EDJ. It can be seen in the model that a group of prisms near the enamel surface runs with prisms approximately parallel to one another, then suddenly divide into two groups running at two different directions as they approach the EDJ possibly giving rise to the two orientation populations of crystallites. Thicker lines in the proposed model represent higher crystallite divergence with respect to one another (Fig. 7).

According to the proposed model in Fig. 7, we suggest that the direction of ameloblast movement may play a role in determining the degree of crystallite orientation; that is, rows of ameloblasts moving in one direction give rise to crystallites with a lower degree of texture (first orientation population), whereas, rows of ameloblasts moving in the opposite direction result in the growth of more highly ordered crystallites (second population of crystallites). Furthermore, the first orientation population was observed to comprise a larger number of crystallites than the second orientation population (Fig. 4a) suggesting that one group of prisms has a higher percentage of crystallites compared to neighboring groups of prisms. The percentage volume of crystallites belonging to each population is represented by the density of lines in the proposed model in Fig. 7.

The small sample size used in this study, due to the scarcity of developing human enamel available for investigation, was a constraint. The other limitation of this study was beamtime allocation. Therefore, even if a large sample set is acquired, it is not guaranteed that sufficient beamtime will be awarded due to the competitive nature of the selection process. Furthermore, the X-ray beam spot size used for this investigation was much greater (50 *μm*) than the reported width of a single prism (≈2–8 *μm*)^2,79,80^ and hence several prisms were averaged in a single diffraction pattern. The useful and novel findings from this study would warrant future studies where it would be insightful to use an X-ray beam with a diameter smaller than a single enamel prism in order to provide more accurate intra-prism data.

Implications of the findings presented in this study include:

- Crystallites in both developing and mature enamel were found to group along two main directions with respect to their *c*-axes, with one population (first orientation population) being more dominant than the other (second orientation population) by a factor of approximately 7:3. To date, no studies have quantified the texture magnitude and direction within two distinct populations of crystallites in developing and mature human enamel. The angular separation between the two populations was found to decrease from the EDJ towards the enamel surface, correlating with a decrease in prism decussation identified using qBSE. Due to this correlation, we propose that the two identified orientation populations may represent the crystallites within the two groups of decussating prisms.
- Crystallites from the two populations in developing and mature enamel were found to orient approximately perpendicular to the enamel surface with an angular separation of 20–50° between the two orientation populations. This suggests that crystallite directions may be defined early in enamel development.
- The texture magnitude values of the two crystallite populations were found to vary in a complex way as a function of maturation stage. Crystallites belonging to the first orientation population displayed considerably lower degree of texture than those from the second population. This arrangement was found to persist from early through to full maturation, supporting the possibility that the texture magnitude is defined during the early stages of enamel biomineralization.
- XMT results served as a confirmation that the mineral content in our archaeological samples followed the expected route of mineralization, progressing from the EDJ peripherally and from the incisal tip cervically as a function of maturation. This indicates that burial for extended periods has had little effect on the mineral content of enamel, giving further validity to our S-XRD results.

## Methods

### Sample selection

Maxillary permanent central incisors were chosen in the current investigation due to the fact that single cusp dentitions are less complex regarding enamel crystal arrangement and therefore offer the simplest case. According to AlQahtani *et al*.^81^, permanent central incisal enamel starts forming approximately 4.5 months after birth and the tooth erupts into the oral cavity between the ages of 6.5–7.5 years after birth. Therefore, in order to gain a complete insight as to how mineralization is progressing, permanent central incisors at various stages of development within the above-mentioned range were required. However, such samples are not readily available for investigation due to the challenges associated with acquiring the necessary ethical approvals. Various studies overcame this issue by recovering developing human enamel samples belonging to deceased juveniles from archaeological sites^29,82–86^. Through this route it was possible to obtain various developing human teeth for comparison. The stage of development was determined according to Alqahtani *et al*.^81^. Teeth were considered to be in early development if the crown was less than three quarters completed; in mid development if the root length was less than the crown length; and in full development if three quarters or more of the root length was developed.

**Figure 8:**
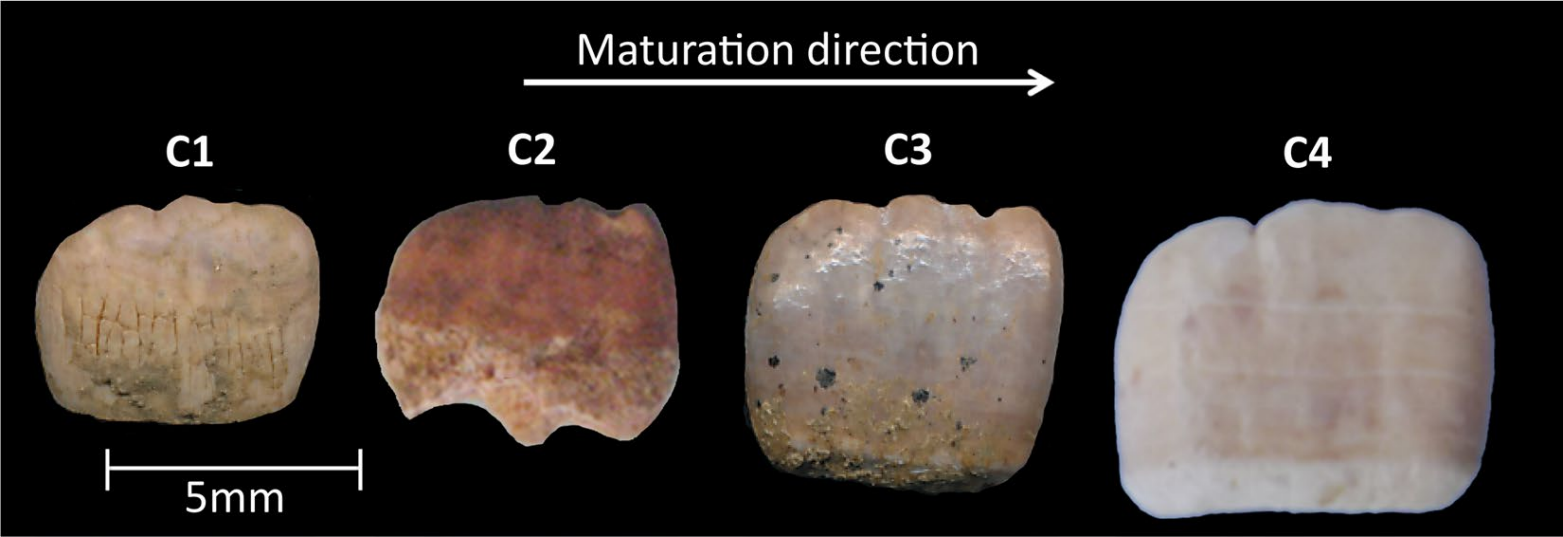
Photographs of the four human permanent maxillary central incisors at various developmental stages obtained from the 12^*th*^–16^*th*^ century AD medieval cemetery of Blackfriars (Gloucester, UK).

Four developing maxillary central incisors (samples C1, C2, C3 and C4) were obtained from the skeletal assemblage excavated from the 12^*th*^–16^*th*^ century AD medieval cemetery of Blackfriars (Gloucester, UK) curated by the Biological Anthropology Research Center, University of Bradford (Bradford, UK) (Fig. 8). A type-matched mature contemporary tooth (sample C5) collected with informed consent from patients treated at Barts and the London Dental Hospital (London, UK) pediatric clinics was used for comparison.

### X-ray microtomography

#### Sample preparation

Whole teeth were analyzed using XMT to quantify and compare mineral concentration variations. Due to the dry nature of archaeological developing teeth (samples C1, C2, C3 and C4), no further sample preparation took place (Fig. 8). The contemporary mature tooth (sample C5) was stored in 100% ethanol solution prior to scanning.

#### Experimental setup

XMT experiments were performed using a fourth generation in-house XMT scanner (MuCAT2) developed at Queen Mary, University of London (QMUL)^42^. The scanner consists of a microfocus X-ray source (X-Tek Systems Ltd., Herts, UK) with 225 *kV* microfocus X-ray generator and 5 *μm* focal spot size, a tungsten target, a kinematic sample stage and an area detector (Spectral Instruments, Tucson, Arizona, USA). The detection system comprises of a cooled 4000 × 4000 charge-coupled devices (CCD) coupled via a parallel fiber-optic faceplate to a columnar caesium iodide (CsI) scintillator (Applied Scintillation Technologies, Essex, UK)^42^.

**Figure 9:**
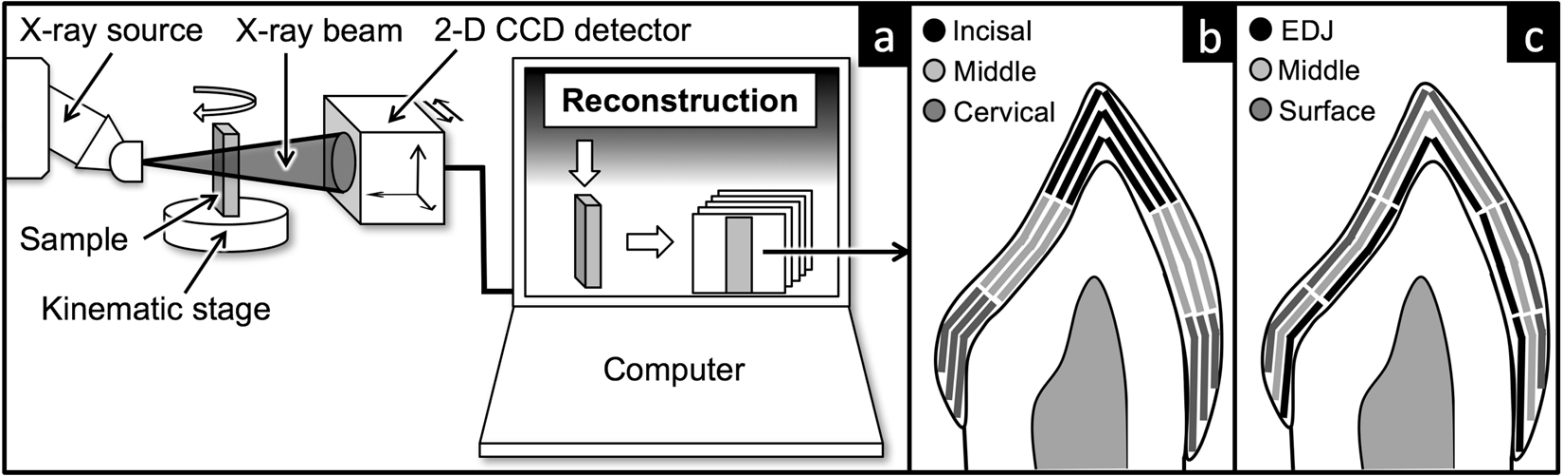
Schematic diagram of (**a**) the XMT experimental setup using the MuCAT 2 scanner (QMUL) and a typical XMT slice showing the 18 regions and how they are divided for (**b**) vertical and (**c**) horizontal analyses.

The CCD and the specimen were mounted on mechanical stages (Physique Instrumente, Palmbach, Karlsruhe, Germany). Time-delay integration CCD readout was employed to eliminate ring artefacts^87^. The spectral spread was minimized using 1.2 *mm* aluminum and 0.05 *mm* copper X-ray filter^42^. In order to correct for beam hardening, each scan was calibrated for 40 *keV* equivalent monochromatic energy using a multi-element calibration carousel with 9 attenuation plates^88^. The samples were scanned with a beam voltage of 90 *kV* and a beam current of 0.18 *mA*. The XMT temperature was maintained at 26 ± 0.1 °*C* to maintain dimensional stability. Cubic voxel resolutions of 15 *μm* and 13 *μm* were set depending on time restrictions. A schematic of the experimental setup is shown in Fig. 9a.

#### Data analysis

The 2-D projections were mathematically reconstructed into stacked 2-D images as a 3-D volume via filtered Feldkamp back-projection algorithm^89^ using an in-house cone beam reconstruction program (ConeRec). Linear attenuation coefficients (LAC) describe the fraction of a beam of X-rays that is absorbed or scattered per unit thickness of the material. In the present study, the LACs of the specimens were calibrated for 40 *keV* equivalent monochromatic energy. The mineral concentration (*x*) at any point along the line profile was determined by:

1 $$x=\frac{{\mu }_{m}}{{\mu }_{p}}\rho $$

Where *μ*_*m*_ is the measured sample LAC, *μ*_*p*_ is the LAC of the pure sample of the mineral and *ρ* is the sample density.

Three software packages were used to analyze the reconstructed data, namely Drishti (Australia National University, Canberra, Australia), ImageJ (Open source, Rashband, 2006) and Tomview (Graham Davis, QMUL, London, UK). Drishti was used mainly for manipulation of 3-D images and differentiation of various features of the dataset. ImageJ is an image processing program that was used to calculate morphometric parameters from reconstructed data and set color scales to mineral concentration values. Tomview, an in-house analysis tool developed by Prof. Graham Davis (QMUL, London, UK), was used to read .*tom* files, display the tomographic dataset in the three Cartesian planes, apply mineral concentration color scales and collect values of LAC expressed in units of *cm*^−1^. A total of 18 regions (9 palatal and 9 labial) were selected for each tooth crown. 200 points were selected from each region and the LAC values were averaged. The average mineral concentration for each region was calculated using Eq. (1). This method allows for analyzing vertical (Fig. 9a) and horizontal (Fig. 9b) mineral concentration.

### Synchrotron X-ray diffraction

#### Sample preparation

**Figure 10:**
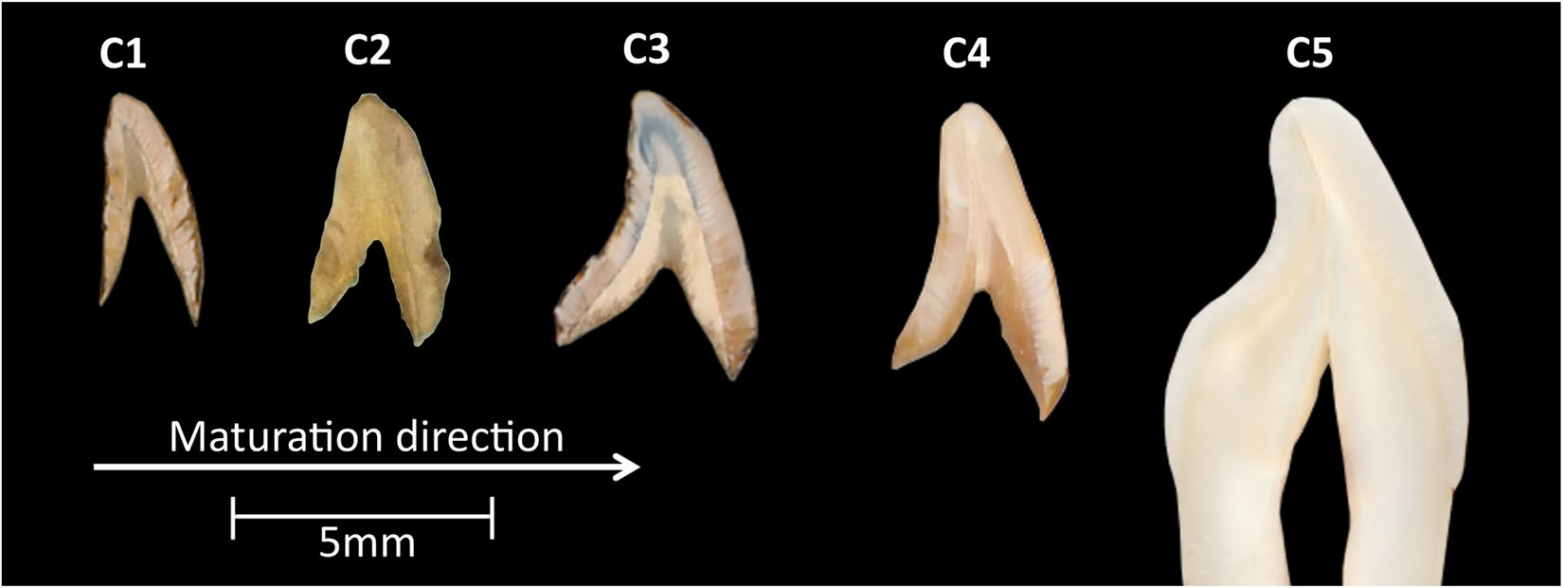
Photographs of the 0.3 mm thick slices of human permanent maxillary central incisors at various developmental stages. For each tooth section, labial is on the right hand side.

The specimens were embedded in fast curing acrylic cold mounting resin (ClaroCit Kit, Steuers, Ballerup, Denmark). This involved placing each tooth in a cylindrical mould. Subsequently, the ClaroCit powder and liquid were mixed as per manufacturer’s instruction, poured into the mould and left to cure for 20 minutes at room temperature. Each of the embedded teeth was cut through the mid-point perpendicular to the bucco-lingual surface using a Struers Accutom-5 diamond saw (Struers, Willich, Germany) to produce 0.3 *mm* thick slice for each tooth (Fig. 10).

#### Experimental setup

**Figure 11:**
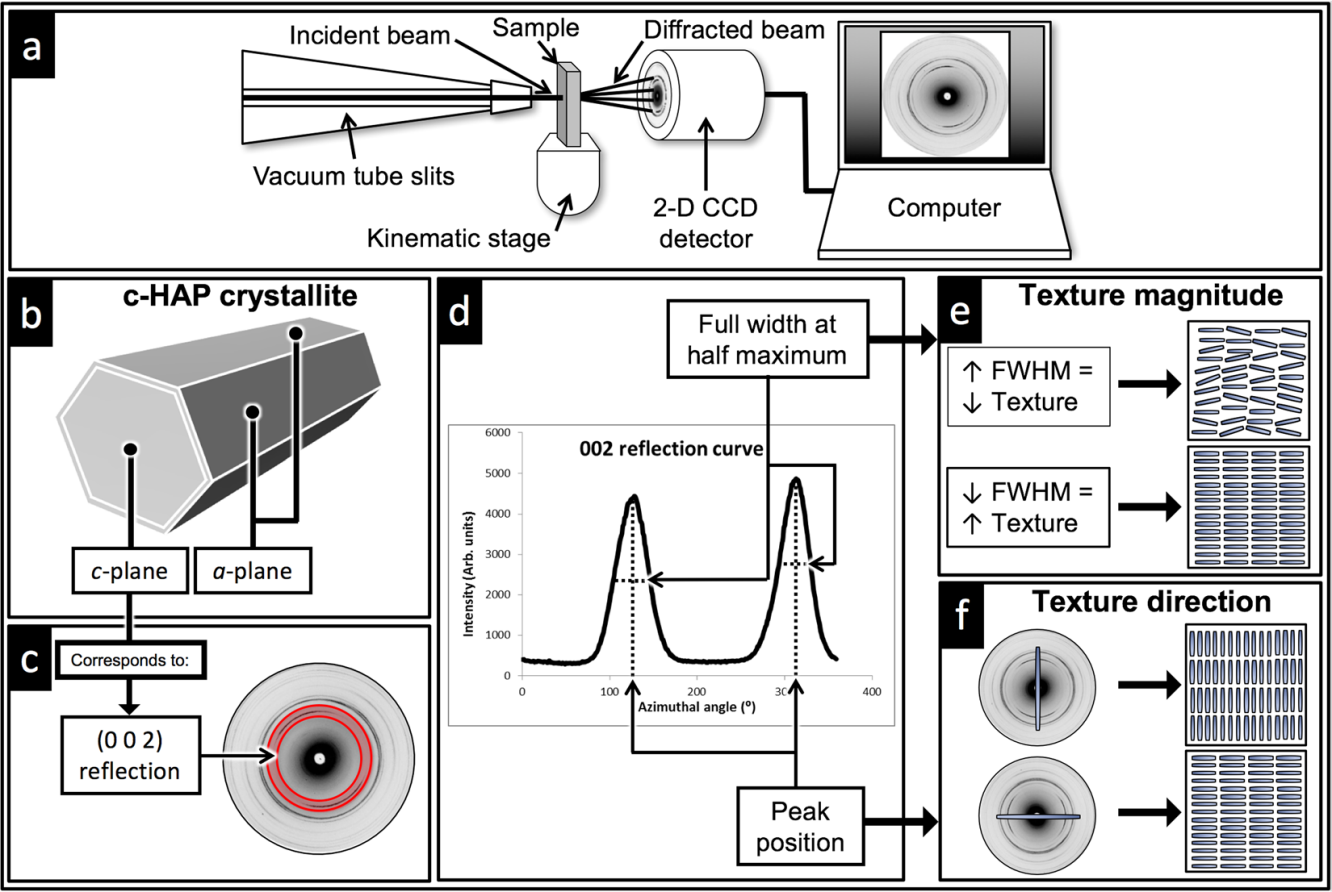
A diagram showing the (**a**) S-XRD experimental setup and the relation between (**b**) the *c*-axis of a c-HAp crystallite and (**c**) the (002) reflection in a typical diffraction pattern. (**d**) The azimuthal 1-D profile of the (002) reflection obtained from the deconvolution of the 2-D diffraction patterns can be used to determine crystalline texture (**e**) magnitude and (**f**) direction.

To satisfy the objectives of this study, experiments were carried out using B16 and XMaS (BM28) beamlines at Diamond Light Source (DLS) and European Synchrotron Radiation Facility (ESRF), respectively. A schematic of the experimental setup is shown in Fig. 11a. The experimental parameters used and a list of the samples analyzed at the two synchrotron beamlines are given in Table 1.

**Table 1:** The experimental parameters used at XMaS and B16 beamlines at ESRF and DLS, respectively.

Beamline	XMaS (BM28)	B16
X-ray energy (*keV*)	15	18
Beam size (*μm*^2^)	50	43
Beam wavelength (*Å*)	0.82	0.69
CCD	MarCCD165 (Rayonix, L.L.C., IL, USA)	ImageStar9000 (Photonic Science Ltd., East Sussex, UK)
CCD resolution (*pixels*)	2048 × 2048	3056 × 3056
CCD pixel size (*μm*^2^)	80	30
Kinematic stage	Huber (Rimsting, Germany)	Newport (California, USA)
Analyzed samples	C1 (palatal), C2 (labial and palatal), C3 (palatal), C4 (labial and palatal), C5 (labial and palatal)	C1 (labial), C3 (labial)

Samples were mounted on a custom built sample holder that was fastened to the beamline kinematic sample stage. The kinematic stages at the two synchrotron facilities allow samples to be scanned at two orthogonal axes perpendicular to the X-ray beam. The 2-D CCD detectors were mounted behind the samples and perpendicular to the incident beam. The dental enamel of each specimen was selected and scanned according to our established methods to produce diffraction images^25^. The sample to detector (S-D) distance was set so that a 2*θ* range of approximately 5–25° could be explored. A total of 18,930 diffraction patterns were collected at *ω* = 0°, i.e. the incident beam is perpendicular to the sample surface. The instrument parameters such as the X-ray wavelength, the sample to detector (S-D) distance and the peak-shape profile were determined using a lanthanum hexaboride (*LaB*_6_) standard sample via the ESRF software package Fit2D^90^. Variations in instrument parameters depended on the operating mode of the synchrotron facilities at the times of the experiments.

When an X-ray beam passes through a sample, it diffracts at the crystallites lattice if the diffraction condition is satisfied for the respective lattice plane (*hkl*). Diffracted rings can be seen on a diffractogram imaged using an area detector. If the illuminated crystallites have a preferred orientation, maxima will appear on these rings. The positions of these maxima contain information regarding the spatial orientation of the corresponding lattice plane (*hkl*)^62^. Fit2D was used to generate composite maps of the CCD diffraction patterns for entire tooth sections. It is established that the (002) lattice plane reflection (2*θ* = 13.71°, highlighted in Fig. 11c), does not overlap with other major reflections and has the greatest variation in intensity with maxima normal to the *c*-axes of the crystallites^29^. Therefore, it is possible to obtain an overview of the directions of preferred orientations for the entire tooth slice by plotting a fiber axis that passes through the centers of the opposing (002) reflection maxima on each diffraction pattern (Fig. 11f) in the composite map^13,22,29^.

The azimuthal 1-D profiles of the (002) reflections were obtained from the deconvolution of the 2-D diffraction patterns using Fit2D. The radial integration width was selected to be close to the width of the (002) reflection (Fig. 11c). The azimuthal profiles were integrated intensity as a function of all azimuthal angles over a narrow band covering the (002) reflection (Fig. 11d). The graph in Fig. 11d displays a typical (002) reflection curve from a sample containing a single preferred orientation. The two pronounced peaks seen in Fig. 11d represent the opposing (002) reflection maxima and are separated by approximately 180°.

**Figure 12:**
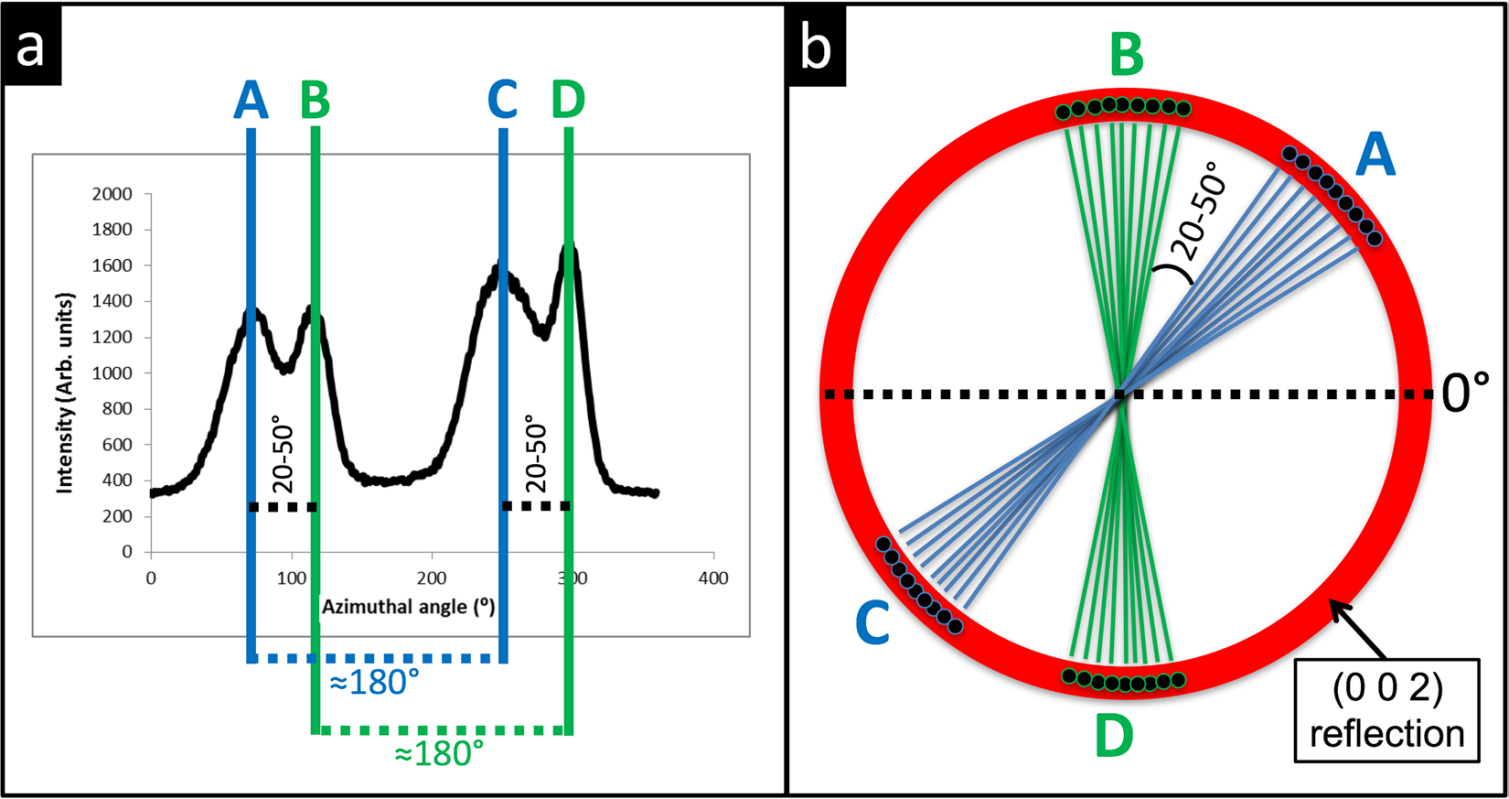
(**a**) A typical (002) reflection curve displaying the variations in intensity around the (002) reflection. Peaks *A* and *C* are separated by approximately 180°, as with peaks *B* and *D*. Peaks *A* and *B* and peaks *C* and *D* are separated by 20–50°. (**b**) A schematic showing the two orientation populations of crystallites, where peaks *A* and *C* represent the first population and peaks *B* and *D* represent the second population with an angular separation of 20–50° between them.

However, the (002) reflection curves obtained during this investigation did not show the expected two pronounced peaks seen in Fig. 11d. Instead, they had either four pronounced peaks or two pronounced peaks each with a shoulder. Spatial analysis of developing and fully developed enamel showed a similar trend regarding the occurrence of these peaks (Fig. 12).

The two additional peaks indicate the presence of two populations of crystallites with distinct preferred orientations. The four peaks were named *A*,*B*,*C* and *D*. Peaks *A* and *C* are separated by approximately 180°, as with peaks *B* and *D*. Peaks *A* and *B* and peaks *C* and *D* are separated by 20–50° (Fig. 12). Therefore, we suggest that peaks *A* and *C* represent the first population and peaks *B* and *D* represent the second population with an angular separation of 20–50° between them (Fig. 12). An in-house automated batching procedure built with MatLab R2017a (MathWorks, Natick, Massachusetts, USA) was used to fit the (002) peaks to a Gaussian peak shape using Eq. (2).

2 $$y=\sum _{i=1}^{n}\,{a}_{i}{e}^{-[{(\frac{x-{b}_{i}}{2{c}_{i}})}^{2}]}$$

Where *a* is the intensity, *b* is the azimuthal angle, *c* is the root mean squared width (RMSW) and *n* is the number of peaks to fit.

**Texture magnitude**: The term texture or preferred orientation is used to describe the organization of crystallites within a polycrystalline material^25–27,29^. The parameter *c* (Eq. (2)) is related to the *FWHM* of the peak according to:

3 $$FWHM=\sqrt{2\,\mathrm{ln}\,2c}$$

FWHM values of peaks *A*(*FWHM*_*A*_) and *C*(*FWHM*_*C*_) and those of peaks *B*(*FWHM*_*B*_) and *D*(*FWHM*_*D*_) were averaged to provide average *FWHM* values for the first (*FWHM*_*pop*1_) and second (*FWHM*_*pop*2_) orientation population, respectively:

4 $$FWH{M}_{pop1}=\frac{FWH{M}_{A}+FWH{M}_{C}}{2}$$

5 $$FWH{M}_{pop2}=\frac{FWH{M}_{B}+FWH{M}_{D}}{2}$$

A lower FWHM value correlates with a higher degree of texture and vice versa (Fig. 11e)^13,22,24,28^.

**Texture direction**: The texture direction was determined using the intensity pattern around the Debye ring of the (002) reflection (Fig. 11c). Parameter *b* from Eq. (2) represents the azimuthal angle and was used to determine the crystallites direction in each diffraction pattern using an automated in-house software built with MatLab R2017a (MathWorks, Natick, Massachusetts, USA). For consistency and clarity, a constant 100 × 100 *μm* spatial resolution was used for each 2-D crystallite direction map with the aid of Kutools for Microsoft Excel v 7.50 (Detong Technology Ltd. Hainan, China). A table of *X* (columns), *Y* (rows) and *Z* (azimuthal angles) values was created for each specimen. Subsequently, a 2-D mesh plot was created were the azimuthal angle value (*Z*) was assigned to each column (*X*) and row (*Z*) point in the mesh in the required order. A 51 × 51 pixels base circular image with a black strip in the middle was defined. The base circular image was set to rotate by the value of each cell in the mesh. A loop was then employed to stack all of the rotated images in a grid that resembles the azimuthal angles seen in the rotation values matrix. A final output image which has as many images as there are cells in the mesh can then be generated.

**Angle between populations**: Parameter *b* from Eq. (2) represents the azimuthal angle and was used to calculate the angular separation between the two orientation populations (Δ_*b*_). Δ_*b*_ was quantified by subtracting the azimuthal angle of peak *B*(*b*_*B*_) from that of peak *A*(*b*_*A*_) and the azimuthal angle of peak *D*(*b*_*D*_) from that of peak *C*(*b*_*C*_) and averaging the two subtracted values:

6 $${{\rm{\Delta }}}_{b}=\frac{({b}_{B}-{b}_{A})+({b}_{D}-{b}_{C})}{2}$$

Δ_*b*_ was calculated for each diffraction pattern and used to construct contour maps using SigmaPlot 10 (Systat Software Inc., San Jose, California, USA).

**Populations percentage**: Parameter *a* from Eq. (2) represents the peak intensity and was used to calculate the percentage of the first orientation population (%_*pop*1_). Peaks intensities of the (002) reflection curve correlate with the quantity of crystallites *c*-planes satisfying the Bragg law. This allows for predicting the relative quantity of each population in every probed region. The intensity of the first orientation population (*a*_*pop*1_) was calculated by averaging the intensities of peaks *A*(*a*_*A*_) and *C*(*a*_*C*_) according to the following equation:

7 $${a}_{pop1}=\frac{{a}_{A}+{a}_{C}}{2}$$

Similarly, the intensity of the second orientation population (*a*_*pop*2_) was calculated by averaging the intensities of peaks *B*(*a*_*B*_) and *D*(*a*_*D*_) according to the following equation:

8 $${a}_{pop2}=\frac{{a}_{B}+{a}_{D}}{2}$$

The values from Eqs (7) and (8) was used to calculate the percentage of the first orientation population (%_*pop*1_):

9 $${ \% }_{pop1}=\frac{{a}_{pop1}}{{a}_{pop1}+{a}_{pop2}}100$$

The %_*pop*1_ values were used to create contour maps using SigmaPlot 10 (Systat Software Inc., San Jose, California, USA).

### Quantitative backscattered electrons imaging

#### Sample preparation

The samples were sectioned as per the method discussed previously. The mid slices of enamel at early- (sample C1), mid- (sample C3) and full-development (sample C5) were assessed using qBSE imaging. The slices were wet-ground with 100-, 220-, 320-, 400- and 600-grit silicon carbide abrasive papers (Buehler, Illinois, USA) on an automatic lapping and polishing unit (Kent 4, Kemet International Ltd., Maidstone, UK). Using successively finer grades of abrasive paper removed damage produced by the earlier grit.

The slices were then polished using a sequence of successively finer particle size diamond polishing pastes ranging from 6 *μm* to 0.25 *μm* (MetPrep, Coventry, UK) to remove the damage imparted by the sawing and grinding operations. Samples were placed in distilled water with 1 *g* of multi-purpose detergent (Teepol, Kent, UK) and ultrasonically cleaned for five minutes using an ultrasonic bath (Kerry PUL-125, Guyson International Ltd., North Yorkshire, UK) between the successive grades of abrasive papers and polishing pastes. The samples were then washed with distilled water and mounted on aluminum stubs. As enamel is electrically non-conductive, a surface conductive coating of evaporated carbon was applied^34^.

#### Experimental setup and data analysis

Microstructural images were collected using Quanta 3-D field emission gun (FEG) dual beam SEM (FEI, Eindhoven, The Netherlands) equipped with a solid-state BSE detector. The SEM was operated at 5 *kV* with ≈10 *mm* working distance. These parameters were kept constant during the taking of BSE micrographs. 9 images at 2400× magnification were obtained from each of samples C3 and C5 and 7 images at the same magnification were obtained from sample C1.

## References

[1] Stack, M. V. Chemical organization of the organic matrix of enamel. In Miles, A. E. W. (ed.) *Structural and Chemical Organization of Teeth*, vol. 2, chap. 20, 317–346 (Academic Press, London, 1967).

[2] Robinson C, Kirkham J, Brookes SJ, Bonass WA, Shore RC (1995). The chemistry of enamel development. Int. J. Dev. Biol..

[3] Elliott, J. C. Structure, crystal chemistry and density of enamel apatites. *In Dental Enamel (Ciba Foundation Symposium)*, vol. 205, 54–72 (John Wiley & Sons Ltd., West Sussex, 1997). 9189617

[4] Elliott, J. C. Hydroxyapatite and nonstoichiometric apatites. In *Structure and Chemistry of the Apatites and Other Calcium Orthophosphates*, vol. 18, chap. 3, 111–189 (Elsevier, Amsterdam, 1994).

[5] Rönnholm E (1962). The amelogenesis of human teeth as revealed by electron microscopy: II. The development of the enamel crystallites. J. ultrastructure research.

[6] Kerebel B, Daculsi G, Kerebel LM (1979). Ultrastructural studies of enamel crystallites. J. Dental Res..

[7] Brown WE, Eidelman N, Tomazic B (1987). Octacalcium phosphate as a precursor in biomineral formation. Adv. dental research.

[8] Dowker SEP, Anderson P, Elliott JC, Gao XJ (1999). Crystal chemistry and dissolution of calcium phosphate in dental enamel. Mineral. Mag..

[9] Beniash E, Metzler RA, Lam RSK, Gilbert P (2009). Transient amorphous calcium phosphate in forming enamel. J. Struct. Biol..

[10] Simmer JP, Richardson AS, Hu Y-Y, Smith CE, Hu JC-C (2012). A post-classical theory of enamel biomineralization... and why we need one. Int. journal oral science.

[11] Daculsi G, Menanteau J, Kerebel LM, Mitre D (1984). Length and shape of enamel crystals. Calcif. Tissue Int..

[12] Berkovitz, B. K. B. *et al*. Teeth. In A. Oksche & Vollrath, L. (eds) *Handbook of Microscopic Anatomy*, vol. 6, chap. Enamel, 309–473 (Springer, Berlin, Heidelberg, 1989).

[13] Glas J-E (1962). Studies on the ultrastructure of dental enamel-II: The orientation of the apatite crystallites as deduced from X-ray diffraction. Arch. Oral Biol..

[14] Boyde, A. *The structure and development of mammalian enamel*. Ph.D. thesis, University of London, London, UK (1964).

[15] Meckel AH, Griebstein WJ, Neal RJ (1965). Structure of mature human dental enamel as observed by electron microscopy. Arch. Oral Biol..

[16] Hirota F (1982). Prism arrangement in human cusp enamel deduced by X-ray diffraction. Arch. Oral Biol..

[17] Hirota F (1986). An explanation for the” two fiber axes” problem in human enamel by X-ray diffraction. J. Dental Res..

[18] Macho GA, Jiang Y, Spears IR (2003). Enamel microstructure—a truly three-dimensional structure. J. Hum. Evol..

[19] Yagi N (2009). Evaluation of enamel crystallites in subsurface lesion by microbeam X-ray diffraction. J. Synchrotron Radiat..

[20] Yagi, N. *et al*. A Microbeam Small-Angle X-ray Scattering Study on Enamel Crystallites in Subsurface Lesion. In *Journal of Physics: Conference Series*, vol. 247 (IOP Publishing, 2010).

[21] Thewlis, J. The Structure of Teeth as Shown by X-ray Examination. *Special Rep. Ser. Med. Res. Counc*. (1940).

[22] Trautz OR, Klein E, Fessenden E, Addelston HK (1953). The interpretation of the X-ray diffractograms obtained from human dental enamel. J. Dental Res..

[23] Poole DFG, Brooks AW (1961). The arrangement of crystallites in enamel prisms. Arch. Oral Biol..

[24] Gwinnett AJ (1966). The ultrastructure of the “prismless” enamel of deciduous teeth. Arch. Oral Biol..

[25] Al-Jawad M (2007). 2D mapping of texture and lattice parameters of dental enamel. Biomater..

[26] Al-Jawad M (2008). Three dimensional mapping of texture in dental enamel. Key engineering materials.

[27] Simmons LM, Al-Jawad M, Kilcoyne SH, Wood DJ (2011). Distribution of enamel crystallite orientation through an entire tooth crown studied using synchrotron X-ray diffraction. Eur. J. Oral Sci..

[28] Al-Jawad M, Addison O, Khan MA, James A, Hendriksz CJ (2012). Disruption of enamel crystal formation quantified by synchrotron microdiffraction. J. Dent..

[29] Simmons LM, Montgomery J, Beaumont J, Davis GR, Al-Jawad M (2013). Mapping the spatial and temporal progression of human dental enamel biomineralization using synchrotron X-ray diffraction. Arch. Oral Biol..

[30] Siddiqui S, Anderson P, Al-Jawad M (2014). Recovery of Crystallographic Texture in Remineralized Dental Enamel. PloS one.

[31] Siddiqui S, Al-Jawad M (2016). Enamelin Directs Crystallite Organization at the Enamel-Dentine Junction. J. Dental Res..

[32] Elliott, J. C., Anderson, P., Boakes, R. & Dover, S. D. Scanning X-ray microradiography and microtomography of calcified tissues. In D. Hukins (ed.) *Calcified Tissue*, 41–63 (Macmillan Education UK, London, 1989).

[33] Gao XJ, Elliott JC, Anderson P, Davis GR (1993). Scanning microradiographic and microtomographic studies of remineralisation of subsurface enamel lesions. J. Chem. Soc..

[34] Fearne JM (1994). Deciduous enamel defects in low-birth-weight children: correlated X-ray microtomographic and backscattered electron imaging study of hypoplasia and hypomineralization. Anat. embryology.

[35] Anderson P, Elliott JC, Bose U, Jones SJ (1996). A comparison of the mineral content of enamel and dentine in human premolars and enamel pearls measured by X-ray microtomography. Arch. Oral Biol..

[36] Davis GR (2002). Non-destructive 3D structural studies by X-ray microtomography. Adv. X-ray Analysis. Newtown Square, Int. Centre for Diffr. Data.

[37] Dowker SEP, Elliott JC, Davis GR, Wassif HS (2003). Longitudinal study of the three-dimensional development of subsurface enamel lesions during *in vitro* demineralisation. Caries Res..

[38] Fearne J, Anderson P, Davis GR (2004). 3D X-ray microscopic study of the extent of variations in enamel density in first permanent molars with idiopathic enamel hypomineralisation. Br. Dental J..

[39] Wong FSL, Anderson P, Fan H, Davis GR (2004). X-ray microtomographic study of mineral concentration distribution in deciduous enamel. Arch. Oral Biol..

[40] Efeoglu N, Wood D, Efeoglu C (2005). Microcomputerised tomography evaluation of 10% carbamide peroxide applied to enamel. J. Dent..

[41] Deyhle H, Bunk O, Muller B (2011). Nanostructure of healthy and caries-affected human teeth. Nanomedicine.

[42] Davis GR, Evershed ANZ, Mills D (2013). Quantitative high contrast X-ray microtomography for dental research. J. Dent..

[43] Rey C, Renugopalakrishnan V, Shimizu M, Collins B, Glimcher MJ (1991). A resolution-enhanced Fourier transform infrared spectroscopic study of the environment of the CO3 2- ion in the mineral phase of enamel during its formation and maturation. Calcif. Tissue Int..

[44] Allan JH (1959). Investigations into the mineralization pattern of human dental enamel. J. Dental Res..

[45] Deutsch D, Gedalia I (1980). Chemically distinct stages in developing human fetal enamel. Arch. Oral Biol..

[46] Avery JK, Visser RL, Knapp DE (1961). The pattern of the mineralization of enamel. J. Dental Res..

[47] Weidmann SM, Weatherell JA, Hamm SM (1967). Variations of enamel density in sections of human teeth. Arch. Oral Biol..

[48] Wilson PR, Beynon AD (1989). Mineralization differences between human deciduous and permanent enamel measured by quantitative microradiography. Arch. Oral Biol..

[49] Applebaum E (1943). Grenz ray studies of enamel matrix formation and calcification. J. Dental Res..

[50] Hals E (1953). Fluorescence microscopy of developing and adult teeth, supplemented by investigations with ordinary, polarizing and phase-contrast microscope. Odontol. Tidskrift.

[51] Engfeldt B, Hammarlund-Essler E (1956). Studies on mineralized denial tissues.: IX. A microradiographic study of the mineralization of developing enamel. Acta Odontol. Scand..

[52] Crabb HSM, Darling AI (1960). The gradient of mineralization in developing enamel. Arch. Oral Biol..

[53] Angmar-Månsson B (1971). A quantitative microradiographic study on the organic matrix of developing human enamel in relation to the mineral content. Arch. Oral Biol..

[54] Suga S (1989). Enamel hypomineralization viewed from the pattern of progressive mineralization of human and monkey developing enamel. Adv. dental research.

[55] Shore, R. C., Robinson, C., Kirkham, J. & Brookes, S. J. Structure of developing enamel. In Robinson, C., Kirkham, J. & Shore, R. (eds.) *Dental Enamel: Formation to Destruction*, 135–150 (CRC Press, New York, 1995).

[56] Skobe Z, Stern S (1980). The pathway of enamel rods at the base of cusps of human teeth. J. Dental Res..

[57] Robinson C (2014). Enamel maturation: a brief background with implications for some enamel dysplasias. Front. Physiol..

[58] Kallistová A, Horáček I, Šlouf M, Skála R, Fridrichová M (2017). Mammalian enamel maturation: Crystallographic changes prior to tooth eruption. PLOS ONE.

[59] Low I (2004). Depth-Profiling of Crystal Structure, Texture and Microhardness in a Functionally Graded Tooth Enamel. J. Am. Ceram. Soc..

[60] Deyhle H (2009). Bio-inspired dental fillings. In Society of Photo-Optical Instrumentation Engineers (SPIE) NanoScience+ Engineering, San Diego, California, USA.

[61] Raue L, Klein H (2011). Calculation of anisotropic properties of dental enamel from synchrotron data. J. Synchrotron Radiat..

[62] Raue L, Gersdorff N, Rödiger M, Klein H (2012). New insights in prism orientation within human enamel. Arch. Oral Biol..

[63] Boyde A (1986). Applications of Tandem Scanning Reflected Light Microscopy and Three-Dimensional Imaginga. Annals New York Acad. Sci..

[64] Boyde, A. Microstructure of enamel. In *Dental enamel (Ciba Foundation Symposium)*, 205, 18–31 (John Wiley & Sons, West Sussex, 1997). 10.1002/9780470515303.ch3 9189615

[65] Robinson C, Brookes SJ, Shore RC, Kirkham J (1998). The developing enamel matrix: nature and function. Eur. J. Oral Sci..

[66] Lyon DG, Darling AI (1957). Orientation of the crystallites in human dental enamel. Br. Dental J..

[67] Glimcher MJ, Daniel EJ, Travis DF, Kamhi S (1965). Electron optical and X-ray diffraction studies of the organization of the inorganic crystals in embryonic bovine enamel. J. ultrastructure research.

[68] Osborn JW (1968). Directions and interrelationship of prisms in cuspal and cervical enamel of human teeth. J. Dental Res..

[69] Lynch CD, O’Sullivan VR, Dockery P, McGillycuddy CT, Sloan AJ (2010). Hunter-Schreger Band patterns in human tooth enamel. J. Anat..

[70] Cuy JL, Mann AB, Livi KJ, Teaford MF, Weihs TP (2002). Nanoindentation mapping of the mechanical properties of human molar tooth enamel. Arch. Oral Biol..

[71] Carlström D (1964). Polarization microscopy of dental enamel with reference to incipient carious lesions. Adv. oral biology.

[72] Beynon AD, Clayton CB, Rozzi FVR, Reid DJ (1998). Radiographic and histological methodologies in estimating the chronology of crown development in modern humans and great apes: a review, with some applications for studies on juvenile hominids. J. Hum. Evol..

[73] Boyde A, Stewart ADG (1963). Scanning electron microscopy of the surface of developing mammalian dental enamel. Nat..

[74] Hirota F (1989). X-ray crystallographic studies as to the calcification in the Hunter-Schreger bands of human enamel. Jpn. J. Oral Biol..

[75] Osborn JW (1990). A 3-dimensional model to describe the relation between prism directions, parazones and diazones and the Hunter-Schreger bands in human tooth enamel. Arch. Oral Biol..

[76] Skobe Z (2006). SEM evidence that one ameloblast secretes one keyhole-shaped enamel rod in monkey teeth. Eur. J. Oral Sci..

[77] Cox BN (2013). How the tooth got its stripes: patterning via strain-cued motility. J. The Royal Soc. Interface.

[78] Koenigswald Wv, Rensberger JM, Pretzschner HU (1987). Changes in the tooth enamel of early Paleocene mammals allowing increased diet diversity. Nat..

[79] Dorozhkin SV (2007). Calcium orthophosphates. J. materials science.

[80] Reyes-Gasga J (2013). XRD and FTIR crystallinity indices in sound human tooth enamel and synthetic hydroxyapatite. Mater. Sci. Eng. C.

[81] Alqahtani SJ, Hector MP, Liversidge HM (2010). Brief communication: the London atlas of human tooth development and eruption. Am. J. Phys. Anthropol..

[82] Peretz B, Nevis N, Smith P (1997). Morphometric variables of developing primary maxillary first molar crowns in humans. Arch. Oral Biol..

[83] Reid DJ, Beynon AD, Rozzi FVR (1998). Histological reconstruction of dental development in four individuals from a medieval site in Picardie, France. J. Hum. Evol..

[84] Zeygerson T, Smith P, Haydenblit R (2000). Intercusp differences in enamel prism patterns in early and late stages of human tooth development. Arch. Oral Biol..

[85] Antoine, D. *Evaluating the periodicity of incremental structures in dental enamel as a means of studying growth in children from past human populations*. Ph.D. thesis, University College London, London, UK (2000).

[86] Mahoney P (2012). Incremental enamel development in modern human deciduous anterior teeth. Am. J. Phys. Anthropol..

[87] Davis GR, Elliott JC (1997). X-ray microtomography scanner using time-delay integration for elimination of ring artefacts in the reconstructed image. Nucl. Instruments Methods Phys. Res. Sect. A.

[88] Evershed, A. N. Z., Mills, D. & Davis, G. Multi-species beam hardening calibration device for X-ray microtomography. In *SPIE Optical Engineering*+ *Applications*, 85061N–85061N–12 (International Society for Optics and Photonics, 2012).

[89] Feldkamp LA, Goldstein SA, Parfitt MA, Jesion G, Kleerekoper M (1989). The direct examination of three-dimensional bone architecture *in vitro* by computed tomography. J. Bone Miner. Res..

[90] Hammersley AP (1997). FIT2D: an introduction and overview. Eur. Synchrotron Radiat. Facil. Intern. Rep. ESRF97HA02T.

